# HENMT1 and piRNA Stability Are Required for Adult Male Germ Cell Transposon Repression and to Define the Spermatogenic Program in the Mouse

**DOI:** 10.1371/journal.pgen.1005620

**Published:** 2015-10-23

**Authors:** Shu Ly Lim, Zhi Peng Qu, R. Daniel Kortschak, David M. Lawrence, Joel Geoghegan, Anna-Lena Hempfling, Martin Bergmann, Christopher C. Goodnow, Christopher J. Ormandy, Lee Wong, Jeff Mann, Hamish S. Scott, Duangporn Jamsai, David L. Adelson, Moira K. O’Bryan

**Affiliations:** 1 Department of Anatomy and Developmental Biology, Monash University, Clayton, Victoria, Australia; 2 School of Molecular and Biomedical Science, University of Adelaide, Adelaide, South Australia, Australia; 3 Australian Cancer Research Foundation Cancer Genomics Facility, Centre for Cancer Biology, SA Pathology, Adelaide, South Australia, Australia; 4 Institute of Veterinary Anatomy, Histology and Embryology, Justus Liebig University Giessen, Giessen, Germany; 5 Australian Phenomics Facility, The Australian National University, Canberra, Australian Capital Territory, Australia; 6 The Garvan Institute of Medical Research, Sydney, Darlinghurst, New South Wales, Australia; 7 The Department of Biochemistry and Molecular Biology, Monash University, Clayton, Victoria, Australia; 8 Murdoch Childrens Research Institute, The Royal Children’s Hospital, Parkville, Victoria, Australia; 9 Department of Molecular Pathology, Centre for Cancer Biology, SA Pathology, Adelaide, South Australia, Australia; University of Cambridge, UNITED KINGDOM

## Abstract

piRNAs are critical for transposable element (TE) repression and germ cell survival during the early phases of spermatogenesis, however, their role in adult germ cells and the relative importance of piRNA methylation is poorly defined in mammals. Using a mouse model of HEN methyltransferase 1 (HENMT1) loss-of-function, RNA-Seq and a range of RNA assays we show that HENMT1 is required for the 2’ O-methylation of mammalian piRNAs. HENMT1 loss leads to piRNA instability, reduced piRNA bulk and length, and ultimately male sterility characterized by a germ cell arrest at the elongating germ cell phase of spermatogenesis. HENMT1 loss-of-function, and the concomitant loss of piRNAs, resulted in TE de-repression in adult meiotic and haploid germ cells, and the precocious, and selective, expression of many haploid-transcripts in meiotic cells. Precocious expression was associated with a more active chromatin state in meiotic cells, elevated levels of DNA damage and a catastrophic deregulation of the haploid germ cell gene expression. Collectively these results define a critical role for HENMT1 and piRNAs in the maintenance of TE repression in adult germ cells and setting the spermatogenic program.

## Author Summary

Piwi-interacting RNAs (piRNAs) are small non-coding RNAs found in great abundance within both embryonic and adult male germ cells. Within embryonic germ cells, piRNAs have a well-recognized role in transposable element (TE) silencing, however, their role in adult cells remains poorly defined. Here we demonstrate that HENMT1 dysfunction and the resultant piRNA instability dramatically impacts multiple aspects of adult germ cell biology. Specifically, pachytene piRNAs are required to maintain TE silencing in adult germ cells and to set the spermatogenic gene expression program. piRNA loss leads to a more active chromatin state in the regulatory regions of numerous normally haploid germ cell genes and their precocious expression during meiosis, followed by a catastrophic deregulation of gene expression in haploid cells and male sterility.

## Introduction

Maintenance of genome integrity in germ cells is crucial. Genomic damage may lead to sterility or disease in offspring and in most animal species is prevented by a number of mechanisms including the silencing of autonomously replicating sequences such as transposable elements (TEs) [1]. TEs are mobile DNA elements which in an unrepressed state have the ability to move from one part of the genome to another, thus inducing mutations and or gene regulatory changes [2]. In order to ameliorate this risk, most species protect their genome in several ways; one such mechanism involves the piRNAs.

piRNAs are 23–32 nucleotide (nt) single stranded RNAs predominantly, but not exclusively, found in the germ line [3,4]. They are derived from intergenic or mRNA 3’UTR [4–6]. In mammals there are two types of piRNAs: pre-pachytene and pachytene piRNAs. Pre-pachytene piRNAs are predominantly expressed in prospermatogonia and are processed by the Argonaute proteins MIWI2 and MILI, including through an amplification loop termed the ping-pong cycle [7]. The majority of pre-pachytene piRNA are TE-derived and involved in TE silencing during early spermatogenesis [7]. PiRNA mediated silencing can occur at both a transcriptional level, as indicated by changes in methylation state, and post-transcriptionally [7–12]. Defects in pre-pachytene piRNA production result in male sterility characterized by TE de-repression and germ cell death in early meiosis [8–11].

Pachytene piRNAs are, however, the more abundant class in the mammalian adult testis [12] where they are found predominantly in meiotic and haploid germ cells. In *Drosophila* it is proposed that piRNA processing occurs by the endonucleolytic cleavage of long precursor transcripts (the primary processing pathway) by the endonuclease Zucchini (Zuc) [13–15]. The equivalent enzyme in mice is PLD6 [14,16,17]. In the mouse testis, piRNA precursors are loaded onto the Argonaute proteins MILI (predominantly found in early germ cells) or MIWI (spermatocytes and spermatids) with a strong bias for uridine at the 5’ end, and trimmed from the 3’ end by an unknown 3’ to 5’ exonuclease. In numerous species piRNAs are then 2’-O-methylated at their 3’ end by an RNA methyltransferase HENMT1 (aka HEN1) [18,19]. The aim of this study is to define the functional importance of piRNA 2’-O-methylation in mammalian (mouse) male fertility and the specific consequences of piRNA loss-of-function in adult germ cell populations.

In plants, the 3’ ends of miRNAs and siRNAs are 2’-O-methylated by the methyltransferase HEN1. In vertebrates, as a consequence of differences in the miRNA processing pathway to that seen in plants, however, only piRNAs are 2’-O-methylated [3,20–22]. The absence of HEN1 (*Pimet*) activity in flies resulted in the loss of piRNA and siRNAs 2’-O-methylation but with no overt phenotypic consequences [22,23]. In contrast, the loss of *hen1* in zebrafish resulted in reduced piRNA content in oocytes, exonuclease-mediated piRNA shortening, and ultimately oocyte loss and female sterility [24]. These studies suggest a role for HEN1 in stabilizing piRNAs in the germ line, but with species-specific consequences. Within mammals the *in vivo* function of the HEN1 orthologue, HENMT1 is undefined, however *in vitro* evidence has indicated a capacity to 2’-O-methylate synthetic piRNAs [25].

In order to define the function of HENMT1 in mammals we examined a *Henmt1* point mutant mouse line. *Henmt1* ^*PIN/PIN*^ males were sterile as a consequence of abnormal haploid germ cell development. At a molecular level HENMT1 dysfunction led to the loss of piRNA methylation, piRNA instability, and TE de-repression in both meiotic and haploid male germ cells. *Henmt1* ^*PIN/PIN*^ male mice also precociously expressed haploid germ cell genes in meiosis, associated with an altered chromatin state in 5’ regulatory regions. These data reveal a critical role for HENMT1 in piRNA metabolism and a role for piRNAs in setting the male germ cell developmental program.

## Results

### HENMT1 is essential for male fertility

**Fig 1:**
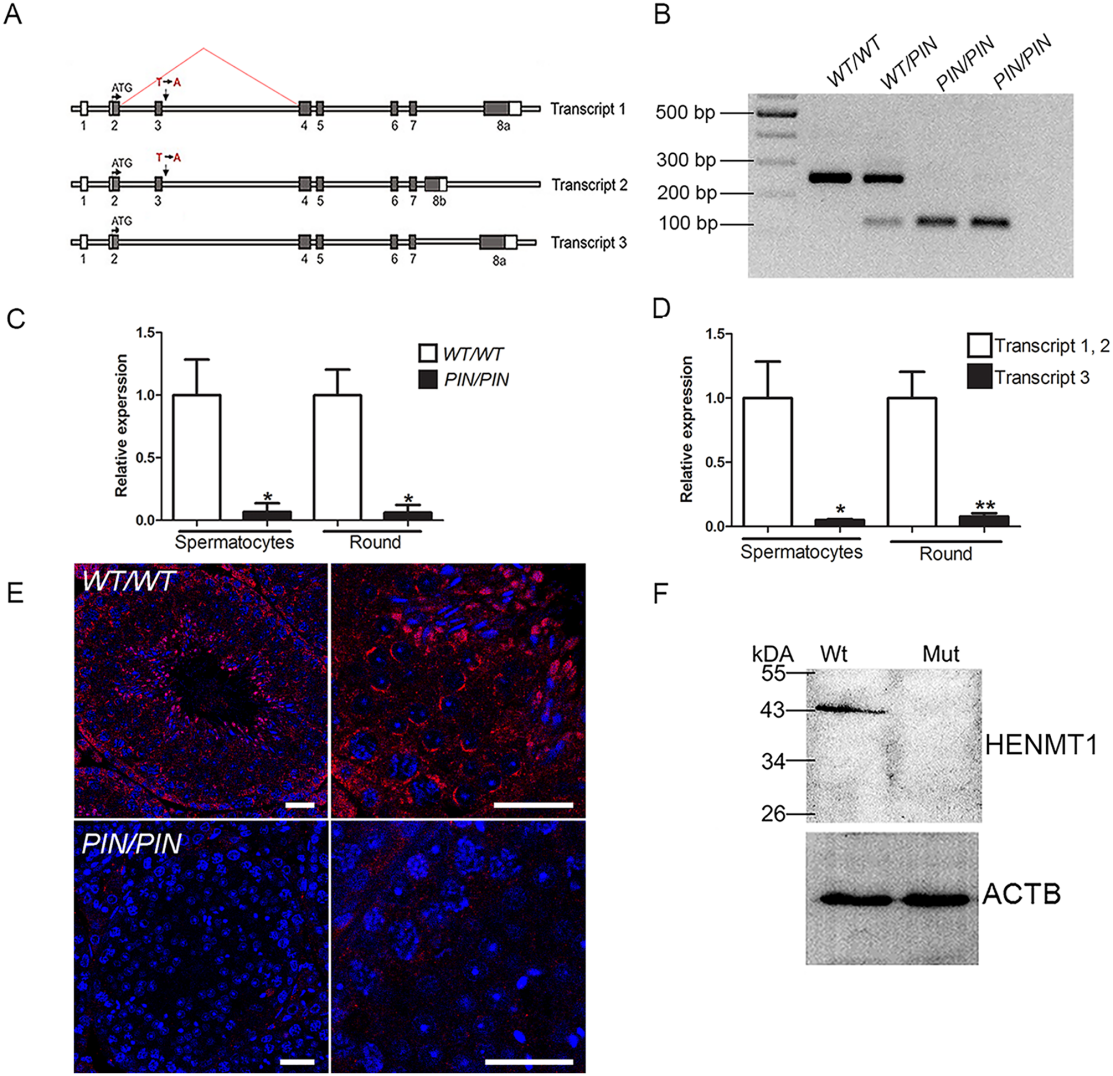
The *Henmt1* ^*PIN/PIN*^ mutation resulted in exon 3 skipping, the production of unstable *Henmt1* mRNAs and an absence of HENMT1 protein in the testis. **(A)** A schematic representation of the three *Henmt1* transcripts and the effect of the *Henmt1* ^*PIN/PIN*^ on exon 3 splicing. Boxes represent exons, open bars represent introns. **(B)** RT-PCR analysis using primers located in exons 2 and 4 confirmed the skipping of exon 3 in the *Henmt1* ^*PIN*^ allele. **(C)** qPCR analysis of *Henmt1* transcript 1 and 2 levels in *Henmt1* ^*PIN/PIN*^ and *Henmt1* ^*WT/WT*^ spermatocytes and round spermatids (round). 8 weeks old, n = 3 /genotype (* p<0.05, mean ± SD). **(D)** The relative expression of *Henmt1* transcripts 1 and 2, and transcript 3 in *Henmt1* ^*WT/WT*^ spermatocytes and round spermatids. 8 weeks old. n = 3 /genotype (p*<0.05, ** p<0.01, mean ± SD). **(E)** HENMT1 localization (red) in *Henmt1* ^*WT/WT*^ (upper two panels) and 8 week old *Henmt1* ^*PIN/PIN*^ testes (lower two panels). DNA is labelled with DAPI (blue). **(F)** A representative western blot for HENMT1 in 8 week old *Henmt1* ^*WT/WT*^ and *Henmt1* ^*PIN/PIN*^ testes. Beta actin was used as a loading control. Where relevant a two-tailed unpaired student T-test was performed for statistical analyses. Scale bars = 50μm.

In order to define the function of HENMT1 in piRNA metabolism and fertility, an N-ethyl-N-nitrosourea (ENU) mutated mouse containing a loss-of-function mutation in the *Henmt1* gene was studied. This mouse line was named ‘Pinhead (*Henmt1* ^*PIN*^)’ by virtue of the possession of highly condensed pin-like sperm heads in the testis. As outlined below *Henmt1* ^*PIN/PIN*^ males were sterile. The Pinhead line contained a single T to A substitution in the conserved GT splice donor site at the 5’ end of intron 3 in the *Henmt1* gene (*Henmt1* ^*PIN*^) (Fig 1A). This substitution was predicted, and subsequently confirmed, to result in the splicing out of exon 3 (exon 3 skipping) and the in-frame deletion of 43 amino acids from the putative methyltransferase domain in two of the three predicted *Henmt1* splice variants (Fig 1A and 1B). RNA-Seq data confirmed exon 3 skipping occurred in virtually all transcripts within *Henmt1* ^*PIN/PIN*^ germ cells (see Materials and Methods for a link to the online raw data). To assess if the *Henmt1* ^*PIN/PIN*^ allele had an effect on *Henmt1* mRNA stability, quantitative PCR (qPCR) was performed using spermatocyte and round spermatid RNA. *Henmt1* transcript 1 and 2 expression (ENSMUST00000059946 and ENSMUST00000098680) were reduced by 93% and 94% in *Henmt1* ^*PIN/PIN*^ spermatoctyes and spermatids respectively, compared to *Henmt1* ^*WT/WT*^ cells (Fig 1C). Transcript 3 (GRCm38/mm10), which does not contain exon 3 and is a minor transcript in the wild type testis (Fig 1D), was up-regulated in *Henmt1* ^*PIN/PIN*^ spermatocytes and round spermatids (S1A Fig), but did not result in discernable protein in either *Henmt1* ^*WT/WT*^ or *Henmt1* ^*PIN/PIN*^ tissue (Fig 1E and 1F). Western blotting suggests that isoform 1 (49kDa) is predominant isoform present in the wild type mouse testis and the *Henmt1* ^*PIN/PIN*^ testes contained no discernable HENMT1 protein (Fig 1F). These data indicate that the Pinhead phenotype is the consequence of a loss of HENMT1 function.

### *Henmt1* is highly testis-enriched

In accordance with a previous publication, our tissue survey revealed that *Henmt1* expression was highly testis-enriched (S1B Fig) [25]. Such an expression pattern was consistent with the absence of pathology in young adult *Henmt1* ^*PIN/PIN*^ mice outside of the testis. An analysis of *Henmt1* expression at key time points during the establishment of the first wave of spermatogenesis showed that *Henmt1* expression was present at all ages but increased dramatically from day 0 to day 18 and to full spermatogenesis (>35 days of age, S1C Fig), consistent with predominant expression in spermatocytes and spermatids. This conclusion was confirmed by immunohistochemistry using a HENMT1-specific antibody (Fig 1E).

### The consequences of HENMT1 dysfunction on testis histology and sperm quality

**Fig 2:**
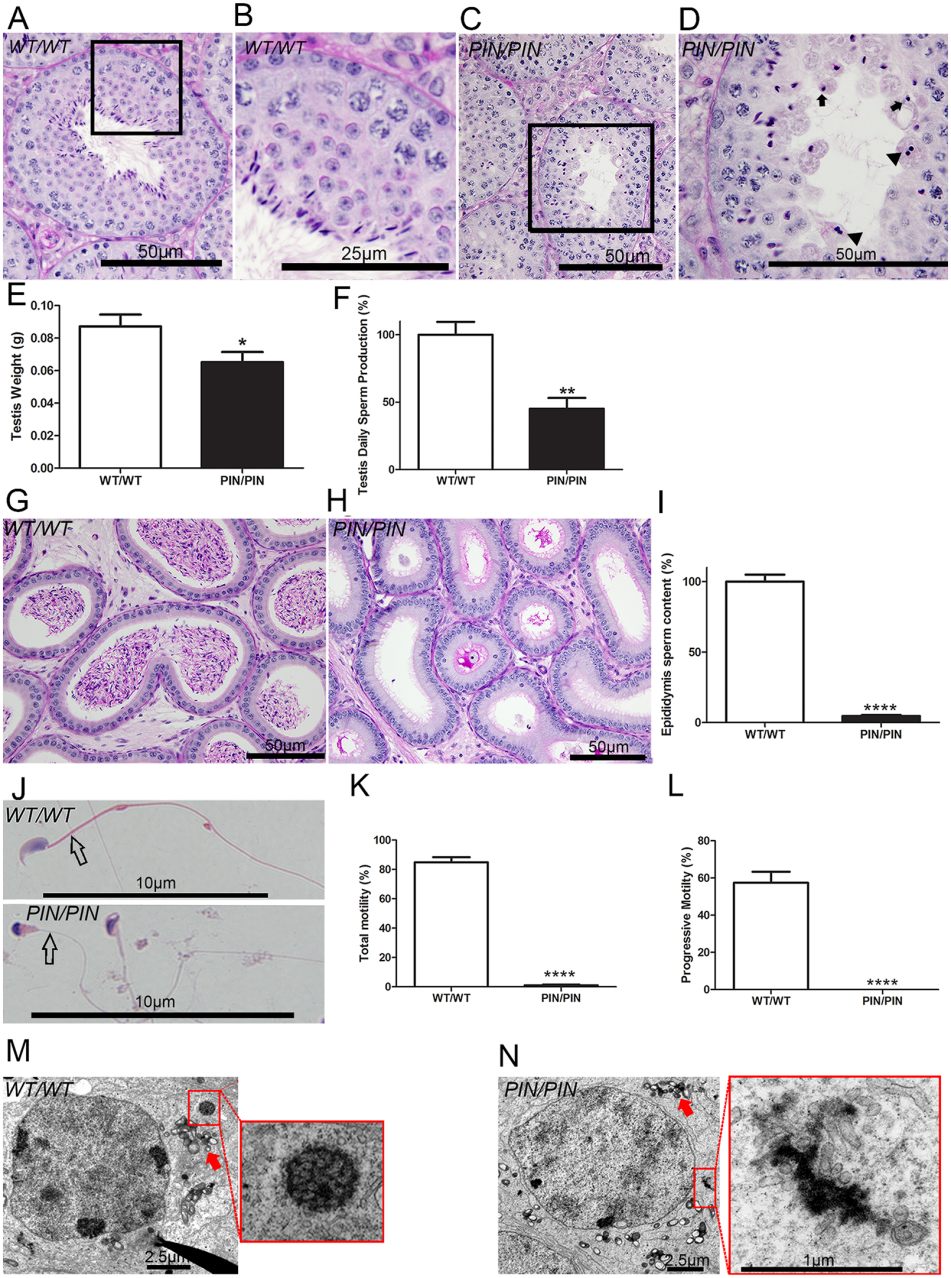
HENMT1 is required for mouse spermatogenesis. **(A-D)** Periodic acid-Schiff (PAS) stained testis sections from *Henmt1* ^*WT/WT*^ and *Henmt1* ^*PIN/PIN*^ mice. Arrows indicate the presence of pinhead-shaped sperm heads. Arrow heads indicate symplasts composed of coalesced germ cells. **(E)** Testis weight in *Henmt1* ^*WT/WT*^ and *Henmt1* ^*PIN/PIN*^ mice (n = 6 / genotype +/- SEM, * p<0.05). **(F)** Daily sperm production in *Henmt1* ^*WT/WT*^ and *Henmt1* ^*PIN/PIN*^ mice expressed as percentage of *Henmt1* ^*WT/WT*^ (n = 6 / genotype +/- SEM, ** p<0.01) **(G-H)** PAS stained sections of epididymides from *Henmt1* ^*WT/WT*^ and *Henmt1* ^*PIN/PIN*^ mice. **(I)** Epididymal sperm content in *Henmt1* ^*WT/WT*^ and *Henmt1* ^*PIN/PIN*^ mice expressed as percentage of *Henmt1* ^*WT/WT*^ (n = 3 / genotype +/- SD, ****p<0.0001). **(J)** Hematoxylin and eosin stained sperm from *Henmt1* ^*WT/WT*^ and *Henmt1* ^*PIN/PIN*^ mice showing the pin-shaped heads and the absence of a mitochondrial sheath (open arrow) in *Henmt1* ^*PIN/PIN*^ sperm. **(K)** The percentage of sperm showing any forms of motility in *Henmt1* ^*WT/WT*^ and *Henmt1* ^*PIN/PIN*^ mice (n = 3 / genotype +/- SD, ****p<0.0001). **(L)** The percentage of sperm showing progressive motility in *Henmt1* ^*WT/WT*^ and *Henmt1* ^*PIN/PIN*^ mice (n = 3 / genotype +/- SD, ****p<0.001). **(M-N)** Electron microscopy of stage VIII pachytene spermatocytes showing the presence of inter-mitochondrial cement (arrow) and the chromatoid body (red square) in *Henmt1* ^*WT/WT*^ and *Henmt1* ^*PIN/PIN*^ mice. All data was collected from 10 week-old mice. A two-tailed unpaired student T-test was performed for statistical analyses.

All *Henmt1* ^*PIN/PIN*^ males were sterile. Heterozygous males and *Henmt1* ^*PIN/PIN*^ females were fertile. Histological analyses at a light microscopic level revealed that *Henmt1* ^*PIN/PIN*^ spermatogenesis occurred apparently normally up to the round spermatid period of development. In the more mature elongate spermatid population, however, there was pronounced germ cell loss and the presence of symplasts composed of coalesced germ cells (Fig 2A–2D). These defects led to a 20% reduction in *Henmt1* ^*PIN/PIN*^ testis weight (Fig 2E) and a 50% reduction in daily sperm output compared to *Henmt1* ^*WT/WT*^ littermates (Fig 2F). Consistent with this presentation the epididymides of *Henmt1* ^*PIN/PIN*^ males contained less than 10% of the number of sperm in *Henmt1* ^*WT/WT*^ epididymides (Fig 2G–2I). The apparent discordance in sperm content between the testis and the epididymis was suggestive of a failure to release sperm (spermiation). This conclusion was confirmed by the presence of retained spermatids in testis sections. Of those sperm that were found in the epididymis, all were abnormal. They possessed stumpy tails and pin-shaped heads (Fig 2J) and had no capacity for progressive motility (Fig 2K and 2L). Consistent with this observation, sperm from *Henmt1* ^*PIN/PIN*^ males lacked a mitochondrial sheath in the mid-piece of the sperm tail (Fig 2J and S2C and S2D Fig). For example, an analysis of spermatogenesis using electron microscopy revealed abnormal accumulations of mitochondria starting in step 10 elongating spermatids (S2A and S2B Fig). By step 15–16 mitochondria were irregularly arranged around the axoneme (S2C and S2D Fig). As such, at a cellular level *Henmt1* ^*PIN/PIN*^ males were sterile as a consequence of a very severe reduction in sperm output, and of those sperm that were produced, all were morphologically abnormal and unable to ascend the female reproductive tract following mating. This phenotype did not worsen with age. The equivalent clinical presentation in humans is known as oligoasthenoteratospermia [26]. We also note that the same phenotype was observed following the backcrossing of the *Henmt1* ^*PIN*^ mutation onto a C57BL6J background, again supporting the causal genotype-phenotype relationship. The results presented here are for the mixed C57BL6J/CBA background.

An electron microscopic analysis of the ultrastructure of *Henmt1* ^*PIN/PIN*^ spermatids revealed development occurred relatively normally up to step 8 elongating spermatids. After this point, abnormalities in the manchette (S2E and S2F Fig) and nuage (Fig 2M and 2N) were observed. The manchette is a transient microtubule structure involved in the shaping of the sperm head and in the transport of proteins into the distal cytoplasm [27]. While the manchette appeared to form normally in *Henmt1* ^*PIN/PIN*^ step 9 spermatids, the contact between the manchette perinuclear ring and acrosome was disturbed resulting in the frequent ectopic positioning of the manchette e.g. towards the plasma membrane rather than the acrosome (S2E Fig). In later steps the formation of the microtubule fringe of the manchette appeared normal, but the perinuclear ring failed to move distally and therefore likely contributed to the observed nuclear constriction (S2F Fig). From step 13 onwards, *Henmt1* ^*PIN/PIN*^ spermatids showed a large heterogeneity in nuclear shape and the degree of DNA condensation.

Analogous to the phenotype observed in several other mouse models containing mutations in piRNA pathway components, the chromatoid body in *Henmt1* ^*PIN/PIN*^ germ cells was abnormal. The chromatoid body is a type of nuage and the site of piRNA metabolism and a proposed site of RNA storage and processing [28]. It is composed of a filamentous and irregular network of anastomosing electron-dense cords surrounded by vesicles. Within *Henmt1* ^*PIN/PIN*^ spermatids the chromatoid body appeared to form normally in step 5 round spermatids (S2G Fig). By step 8, however, when the chromatoid body should have formed a definitive honeycomb shape, the *Henmt1* ^*PIN/PIN*^ chromatoid body was an amorphous mass surrounded by vesicles (Fig 2M and 2N). The equivalent structure in *Henmt1* ^*WT/WT*^ germ cells did not appear until step 11 elongating spermatids, consistent with the premature dissipation of the chromatoid body in *Henmt1* ^*PIN/PIN*^ germ cells. Inter-mitochondrial cement, another type of nuage found in spermatocytes, did not differ significantly between *Henmt1* ^*WT/WT*^ and *Henmt1* ^*PIN/PIN*^ germ cells (S2H Fig).

### HENMT1 regulates mammalian piRNA stability

**Fig 3:**
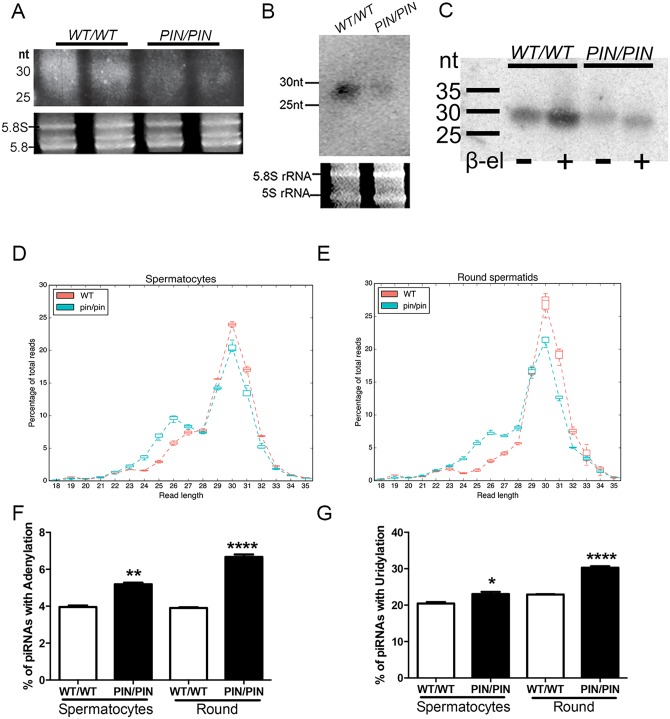
HENMT1 loss results in decreased piRNA bulk and the absence of 3’ 2’-O-methylation and stability. **(A-B)** A representative image illustrating a ~51% reduction in the abundance of piRNAs in *Henmt1* ^*PIN/PIN*^ and *Henmt1* ^*WT/WT*^ 30 day-old testes as indicated on an acrylamide gel (total piRNAs, A) and a small RNA northern blotting for pachytene piRNA1 (B). 5.8S and 5S rRNAs were used as loading controls. Statistics were carried out on n-4 biological replicates per genotype. **(C)** The effects of β-elimination on piRNA length. 5.8S and 5S rRNAs were used as loading controls. **(D-E)** piRNA length distributions, expressed as a percentage of total, in *Henmt1* ^*WT/WT*^ and *Henmt1* ^*PIN/PIN*^. **(F-G)** The percentage of piRNAs with (F) adenylation (last base of read was A when the reference base was not A) and (G) uridylation (last base of read was T, reference base was not T) at their 3’ end in spermatocytes and round spermatids (n = 2 / *Henmt1* ^*WT/WT*^ spermatocytes, n = 3 *Henmt1* ^*WT/WT*^ round, *Henmt1* ^*PIN/PIN*^ spermatocytes and round +/- SD, * p<0.05; ** p<0.01; ***p<0.001; ****p<0.0001). Round = round spermatids. A two-tailed unpaired student T-test was performed for statistical analysis.

In order to test whether HENMT1 functions to add 2’ methyl groups to the 3’ end of mouse piRNA *in vivo* and to ascertain if this function was altered in *Henmt1* ^*PIN/PIN*^ males, total RNA was isolated from 30 day-old *Henmt1* ^*WT/WT*^ *and Henmt1* ^*PIN/PIN*^ testes and abundance assessed. This age was chosen in order to minimise variations in germ cell content between genotypes. A densitometry analysis of piRNA bulk indicated that *Henmt1* ^*PIN/PIN*^ testes compared to *Henmt1* ^*WT/WT*^ contained only 49% of that contained *Henmt1* ^*WT/WT*^ testes (100% ± 10.02 versus 49% ± 12.04, n = 4, p<0.01, Fig 3A). This result was confirmed by small RNA northern analysis for piR-1 (Fig 3B).

To ascertain if the loss of piRNA content was associated with the absence of 3’ methyl groups we performed the β-elimination assay on small RNAs from wild type versus *Henmt1* ^*PIN/PIN*^ testes. In this assay piRNAs lacking a 3’ 2’-O-methyl group will lose their 3’-most base resulting in an apparent size shortening compared to 2’-O-methylated piRNAs [25,29]. As illustrated in Fig 3C, following treatment *Henmt1* ^*PIN/PIN*^ piRNAs were 1 nucleotide shorter than those from *Henmt1* ^*WT/WT*^ piRNAs. Thus, mouse piRNAs are normally 2’-OH-methylated at their 3’ ends *in vivo* and the loss of HENMT1 function leads to a lack of methylation.

To further assess piRNA characteristics in the absence of HENMT1 function we performed deep sequencing of small RNAs in *Henmt1* ^*WT/WT*^ and *Henmt1* ^*PIN/PIN*^ spermatocytes and round spermatids. RNA-Seq resulted in a total of 355M reads with at least 46M reads for each genotype and replicate. The reads were trimmed of adapter sequences with Cutadapt [30] and mapped to the mm10 assembly with BWA [31], with over 95% of reads aligning correctly. piRNAs were defined as being 23–32 nt in length, with primary piRNAs having a 'U' in position 1 and secondary piRNAs having an 'A' in position 10. 80% of reads obtained were contained within previously described piRNA clusters and 83–87% met our sequence-based criteria for piRNAs (S1 Table). Consistent with previous reports [32], 91% of piRNAs in spermatocytes and spermatids mapped to intergenic regions; ~4% mapped to non-coding RNAs; ~4% mapped to protein coding genes and ~0.5% mapped to repeat regions (S3A and S3B Fig). As expected for adult germ cells wherein MIWI is the predominant Argonaute protein, the bulk of piRNAs from *Henmt1* ^*WT/WT*^ cells were 28–32 nt in length. In contrast, those piRNAs that remained in *Henmt1* ^*PIN/PIN*^ spermatocytes and round spermatids contained a reduced peak at 30 nt and a second peak at 26 nt suggestive of piRNA instability (Fig 3D and 3E). These data are indicative of either the shortening of the entire piRNA population or the preferential loss of MIWI-associated (28-32nt) piRNAs. In order to determine which of these two possibilities was occurring we compared MIWI and MILI associated HITS-CLIP data derived from whole mouse testis [33] to our small RNA Seq data. As defined by the HITS-CLIP data MILI-loaded piRNAs accounted for about 7–8% and MIWI-derived piRNA accounted for 10–12% of the total size-selected reads in our wild type and mutant samples (S4A and S4B Fig and S2 Table). We then plotted the size distribution of MILI-loaded or MIWI-loaded piRNAs from wild type versus mutant spermatocytes and round spermatids (S4C and S4D Fig and S2 Table). This analysis revealed the shortening of both MILI and MIWI-derived piRNAs in mutant cells thus strongly suggesting that the loss of HENMT1 function leads to instability of both MILI and MIWI associated piRNAs.

To further explore the precise nature of RNA shortening we examined a variety of piRNA characteristics using the bíogo bioinformatics toolkit. Initially, wild type piRNAs were analysed according to alignment length and genomic location. This involved filtering aligned reads by sequence, mapping quality, and piRNA sequence features to read-length. Filtered reads were then stratified into 10Mbp genomic bins. The results of this analysis were consistent with the total small RNA length distribution shown in Fig 3D and 3E, and largely consistent with the locations of known piRNA loci [34].

A similar analysis to that described above was also conducted but with an additional step of calculating the proportional difference between piRNA lengths in *Henmt1* ^*WT/WT*^ and *Henmt1* ^*PIN/PIN*^ cells (S5A–S5C Fig). This comparison showed that of the remaining piRNAs in *Henmt1* ^*PIN/PIN*^ cells at all piRNA expressing loci, the proportion of short piRNA (23–27 nt) increased and long piRNA (28–32 nt) decreased. In both analyses, long and short piRNA pool expression levels were calculated. In order to determine the numbers of distinct piRNA genus, counts of unique 5' ends were tallied for each bin. As piRNA truncation at the 5' end of piRNAs was infrequent, 5' end count was considered a good proxy for genus count. Collectively these analyses showed that the *Henmt1* ^*PIN/PIN*^ allele resulted in a global shift towards a shorter piRNA population.

To define exactly how piRNA were shortened we compared short piRNA pool alignments with their homologous long pool alignments. This analysis involved assigning each short alignment to the longest alignment that completely contained the short alignment and then determining the degree of truncation at each end required to explain the short alignment assuming that the short piRNA was a truncation product of the long piRNA. The difference between truncation at the 5' and 3' ends of short piRNA alignments was investigated for both cell types and there was a clear difference between the two ends and between genotypes (S6A and S6B Fig). piRNAs were significantly truncated at the 3' end in both spermatocytes and round spermatids (Welch Two Sample t-test, p<2.2x10^-16^).

Notably, these data also revealed a shift in the ratio between 1° and 2° piRNAs in *Henmt1* ^*WT/WT*^ versus *Henmt1* ^*PIN/PIN*^ cells (S7 Fig), an observation that is most parsimoniously explained by the existence of ping-pong amplification of piRNAs in meiotic and post-meiotic male germs cells. For this analysis we used more stringent criteria to define primary and secondary piRNAs and observed that within the remaining piRNAs in *Henmt1* ^*PIN/PIN*^ spermatocytes and round spermatids there was a 40–50% decrease in the proportion of piRNAs in the ping-pong cycle in *Henmt1* ^*PIN/PIN*^ compared to wild type cells (S7A Fig). We then examined the proportion of primary versus secondary piRNAs that mapped to either TEs or protein coding genes (S7B and S7C Fig) and found that piRNAs that mapped to TEs had proportionately more secondary piRNAs in both cell types. Interestingly, piRNAs that mapped to genes showed proportionally more secondary piRNAs in spermatocytes and more primary piRNAs in round spermatids. The Z scores of overlapped piRNA pairs (size-selected reads ranging from 23 to 32 nt) were calculated with signature.py [35]. The ping-pong Z score was defined by 10 nt overlaps for piRNA pairs at the 5’ end [36]. We observed the existence of ping-pong amplification from the Z score plot for all overlapping occurrences (S8A Fig). When we compared the ping-pong Z scores of wild type and mutant samples, we saw decreased ping-pong Z scores in mutant samples compared to wild type in both developmental stages (S8B Fig). This is consistent with what we reported in S7 Fig. While previous reports have suggested there is a weak ping-pong amplification signature in spermatocytes and spermatids [37], our observation of secondary piRNAs that can only result from ping-pong amplification is a new finding.

An analysis of the 3’ end of individual piRNA sequences also revealed a significant increase in the proportion of piRNAs from *Henmt1* ^*PIN/PIN*^ cells that were adenylated or uridylated at their 3’ ends (Fig 3F and 3G) suggesting the active degradation of piRNAs [24,38,39]. Collectively these data reveal greatly reduced piRNA stability in the absence of HENMT1 function. This is the first *in vivo* demonstration that mouse HENMT1 functions in the stabilization of piRNAs in adult mammalian germ cells. Further, these results establish the *Henmt1* ^*PIN/PIN*^ mouse lines as a model of piRNA loss of function.

### HENMT1 is necessary for retrotransposon silencing in spermatocytes and spermatids

**Fig 4:**
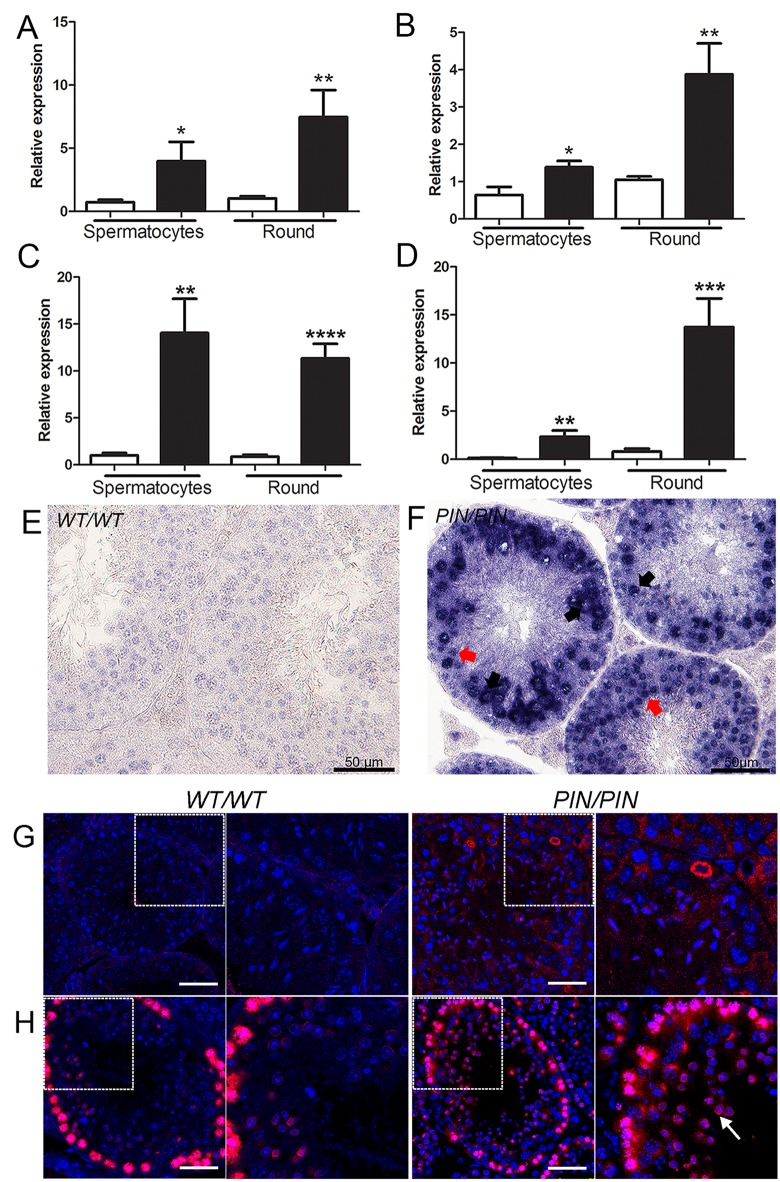
HENMT1 is required for the repression of *Line-1* and *IAP* retrotransposons in spermatocytes and round spermatids. **(A-D)** qPCR analyses of (**A**) *L1_A* (**B**) *L1_TF14* (**C**) *IAP_LTR* (**D**) *IAP_GAG* in *Henmt1* ^*WT/WT*^ and *Henmt1* ^*PIN/PIN*^ spermatocytes and round spermatids (round) purified from 30 day old mice. A two-tailed unpaired student T-test was performed for statistical analysis, n = 5/genotype, +/- SEM, * p<0.05; ** p<0.01; ***p<0.001; ****p<0.0001. **(E-F)** RNA *in situ* hybridisation for *Line-1* expression in *Henmt1* ^*WT/WT*^ and *Henmt1* ^*PIN/PIN*^ 10 weeks-old testes. Black arrows indicate pachytene spermatocytes. Red arrows indicate round spermatids. **(G)** Staining for LINE-1 in in *Henmt1* ^*WT/WT*^ and *Henmt1* ^*PIN/PIN*^ in 10 weeks-old testes. The boxed areas are magnified in the panel to the immediate right of each genotype. The scale bar = 50 μm. (**H**) Immunofluorescence staining for γH2AX (red) as a marker of DNA double stranded break respectively in *Henmt1* ^*WT/WT*^ and *Henmt1* ^*PIN/PIN*^ in 10 weeks-old testes. The white arrow indicates elevated γH2AX staining in spermatids compared to wild type cells. The boxed areas are magnified in the panel to the immediate right of each genotype. The scale bar = 50 μm.

piRNAs are well known for their role in protecting genome integrity via repression of retrotransposon expression in the early phases of spermatogenesis [40]. Mutations in piRNA pathway genes including *Miwi*, *Mael* and *Mov10l1*, lead to the over-expression of retrotransposons in prospermatogonia through to early meiotic cells in the mouse, after which germ cells are lost via apoptosis [9,41–43]. As such, we asked whether the loss of HENMT1 resulted in the derepression of TE in adult germ cell types. *L1* and *IAP* RNA expression were increased in *Henmt1* ^*PIN/PIN*^ spermatocytes and round spermatids (Fig 4A–4D). Elevated L1 expression was confirmed by *in situ* hybridisation on testis sections (Fig 4E and 4F), immunofluorescence (Fig 4G) and the expression of *L1_A* and *L1_TF14* was confirmed by RNA-Seq (S9A Fig). Consistent with elevated TE expression and a previous report showing that L1 ORF2p de-repression lead to increased levels of double stranded breaks (DSB), [44] we observed increased localization of the DSB associated protein γH2AX in *Henmt1* ^*PIN/PIN*^ spermatocytes and spermatids (Fig 4H). These results are suggestive of elevated genome instability following piRNA loss. As such, HENMT1 and pachytene piRNA function is required in order to maintain TE silencing and genome stability in adult male germ cells. Of interest, the *in situ* hybridization data indicates that the degree of TE de-repression was stronger in meiotic and haploid germs cells than in spermatogonia (Fig 4F). This observation is consistent with previously published data suggesting spermatogonial TE repression is maintained in the absence of piRNAs function in adult spermatogeneis [45] and may partially explaining the preferential loss of adult germ cells types over stem cells.

Within prospermatogonia retrotransposons are normally repressed by the piRNA pathway via the recruitment of the DNA methylation machinery, and mutations in key piRNA pathway components lead to retrotransposon de-repression and associated changes in *de novo* DNA methylation [8,10,46]. The association between DNA methylation and TE silencing in later germ cells types is somewhat more controversial [11,45]. In order to explore the necessity for HENMT1, and by extension piRNA function, in genome methylation in adult germ cell populations we performed DNA methylation sensitive Southern blotting using an *L1_A* probe on isolated spermatocytes and round spermatids from *Henmt1* ^*PIN/PIN*^ and *Henmt1* ^*WT/WT*^ testes. Analogous to the situation in *Miwi* null mice [11], we observed no appreciable change in global DNA methylation at the *L1* and *ERVII* sites in the *Henmt1* ^*PIN/PIN*^ spermatocytes and round spermatids (S9B and S9C Fig) indicating a fundamental difference in the mechanism of TE silencing between the foetal and adult germ line. We cannot, however, exclude more subtle changes beyond the detection limits of Southern blotting.

### HENMT1 dysfunction leads to a dys-regulation in the timing of spermiogenic gene expression

**Fig 5:**
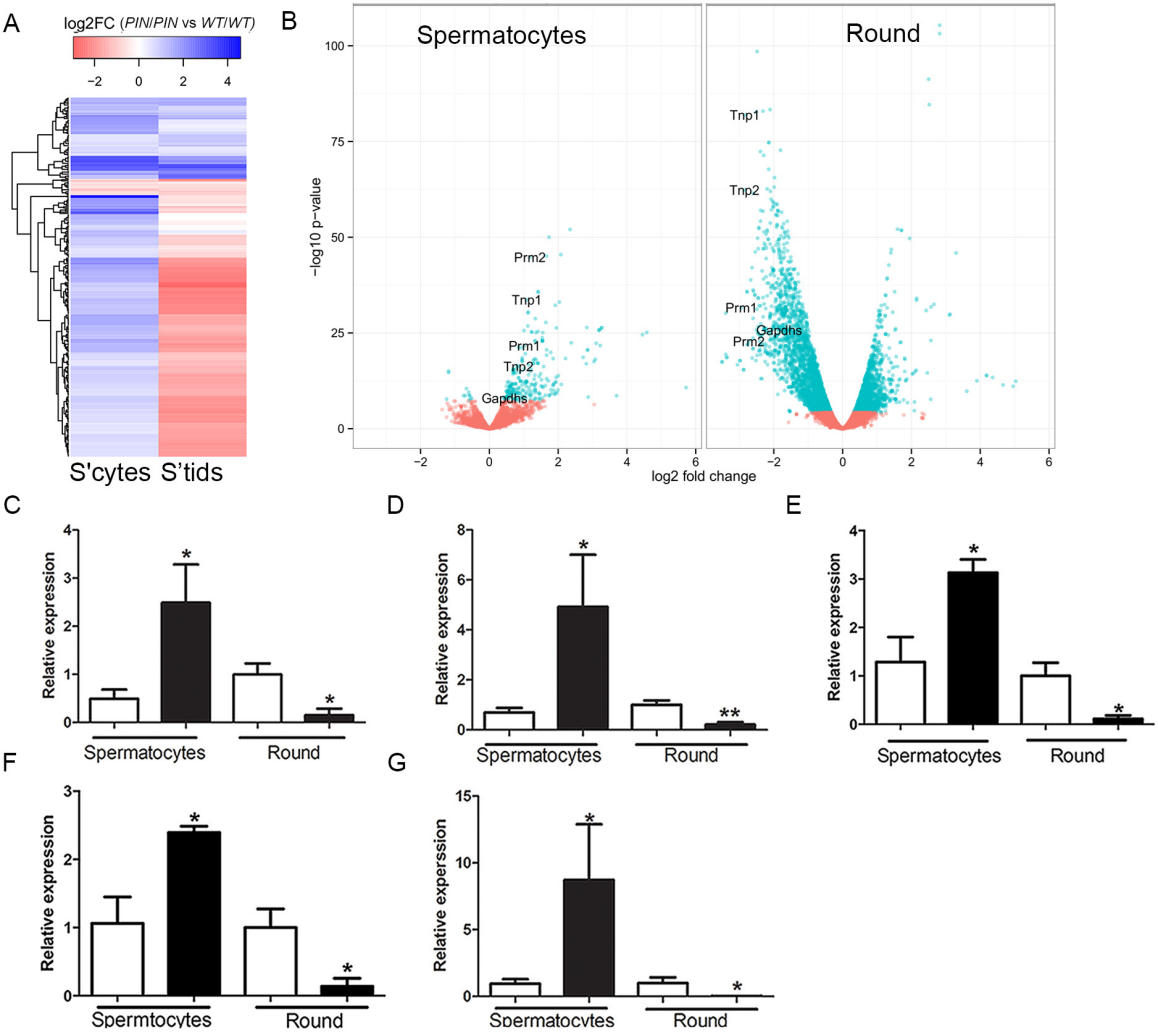
Altered gene expression in *Henmt1* ^*PIN/PIN*^ spermatocytes and round spermatids. **(A)** A heatmap showing log2 fold change in differentially expressed genes in spermatocytes and their corresponding log2 fold change in round spermatids (round). Color coding represent log2 fold changes of genes. *Prm1*, *Tnp1*, *Tnp2*, *Prm2* indicate the position of transcripts subjected to further analysis. **(B)** A volcano plot showing expression changes of all detected genes in spermatocytes and round spermatids. Dots in cyan represent significantly differential expressed genes (FDR < 0.05) based on edgeR. Fold changes and p-values were calculated with edgeR. **(C-G)** qPCR of spermiogenic genes including (C) *Tnp1* (D) *Tnp2* (E) *Prm1* (F) *Prm2* (G) *Gapdhs* in 28 day-old *Henmt1* ^*WT/WT*^ and *Henmt1* ^*PIN/PIN*^ spermatocytes and round spermatids (n = 5 / genotype +/- SEM, * p<0.05, **p<0.01). White bar represents *Henmt1* ^*WT/WT*^ and black bar is *Henmt1* ^*PIN/PIN*^. S’cytes = spermatocytes, S’tids = round spermatids. A two-tailed unpaired student T-test was performed for statistical analyses.

Despite pachytene piRNAs being most abundantly expressed in meiotic and haploid germ cells their role(s) during these periods of spermatogenesis remain largely unknown [47]. We note however, a recent publication revealing a role for piRNAs in the mass elimination of mRNA species via a piRNA-RISC mechanism in elongating spermatids immediately prior to sperm release i.e. after the period wherein germ cells are lost in *Henmt1* ^*PIN/PIN*^ mice [48]. In order to investigate the role of HENMT1, and by extension piRNAs function, in meiotic and round spermatid germ cell gene expression, we performed whole transcriptome analysis on *Henmt1* ^*WT/WT*^ and *Henmt1* ^*PIN/PIN*^ cells. Differences in transcript levels between genotypes were identified with edgeR [49]. Within *Henmt1* ^*PIN/PIN*^ spermatocytes 256 genes were up-regulated and 12 down-regulated compared to *Henmt1* ^*WT/WT*^ (Fig 5A) detected. In contrast within round spermatids 2,386 mRNAs were significantly up-regulated and 3,479 down-regulated compared to *Henmt1* ^*WT/WT*^ (Fig 5B; S1 Dataset). Notably, of the 256 up-regulated genes in spermatocytes, 168 (66%) were subsequently down-regulated in round spermatids and the majority of others were normally expressed at maximal levels in spermatids (Fig 5A and 5B; S1 Dataset). Many of these genes, including *Prm1*, *Prm2*, *Tnp1*, *Tnp2*, *Spem1* and *Gapds*, have previously been shown to have an essential role in haploid germ cell development and male fertility [50–53]. Changes in gene expression for several essential genes were confirmed using qPCR (Fig 5C–5G). Collectively these results were suggestive of the precocious and preferential expression of haploid genes in meiotic cells.

In order to ascertain if particular pathways were being selectively perturbed by the loss of HENMT1 function, we undertook a Gene Ontology (GO) term analysis of differentially expressed genes subgroups between meiotic and haploid cells. This revealed striking differences in proposed function between transcript sets (S1 Dataset). For example, the 256 genes up-regulated in spermatocytes were overwhelmingly associated with germ cell-specific functions including male gamete generation, reproductive developmental processes, germ cell development, nucleosome organization, sperm motility, chromosome organization, cellular macromolecular complex subunit organization, and chromatin assembly (S1 Dataset). Those that were unchanged in meiotic cells but either up- or down-regulated in round spermatids were associated with generalized cell metabolism including cellular macromolecule metabolism, nucleic acid metabolism, and chromosome organization (S1 Dataset). Collectively these data are consistent with the loss of HENMT1 and piRNA function leading to a specific dysregulation of spermatogenesis gene expression during meiosis followed by a secondary and catastrophic failure in global gene regulation in haploid cells, and is congruent with the ultimate loss of elongate spermatids from the seminiferous epithelium (Fig 2A–2D).

*In toto* these results, and the associated histological changes in *Henmt1* ^*PIN/PIN*^ mice, suggest three possible mechanisms which in combination or individually could lead to sterility: 1) the loss of piRNAs directed against ‘spermatid’ transcripts in meiotic cells leads to their relative enrichment i.e. the loss of piRNA-mediated mRNA decay; 2) a loss of translational delay in key haploid germ cell transcripts leading to their depletion in round spermatids; or 3) a change in chromatin structure resulting in the precocious transcription of normally spermatid-expressed genes in meiotic cells. In order to test these possibilities we concentrated on spermatocytes, which had relatively fewer mRNA expression changes and wherein development continued without notable phenotypic consequences. As indicated above by the GO term analysis, many of the expression changes in round spermatids may have been due to secondary effects and associated with the ultimate demise of these cells thus introducing confounding factors.

With respect to the first possibility, we could find no evidence of a statistically significant correlation between lowered piRNA levels and increased mRNA levels (Dataset 1). A similar result was reported by [48] for spermatocytes. Indeed we observed a greater abundance of sense strand-specific piRNAs than anti-sense strand-specific piRNAs in wild type spermatocytes i.e. the majority of spermatocyte piRNAs were not anti-sense to mRNAs and the loss of HENMT1 function did not lead to perturbation of piRNA-mediated mRNA decay in spermatocytes.

In relation to a possible role for piRNAs in translational delay, two of the most critical processes that occur during spermiogenesis are the condensation of the haploid genome and the development of the sperm tail. The mRNAs involved in these processes are unusual in that the majority undergo a period of translational delay during which mRNAs are stored in ribonucleoprotein complexes, often for days, prior to translation [54,55]. The biological necessity for this delay is the exchange of histones for transition proteins and ultimately protamines, and the progressive condensation of the nucleus such that it becomes inaccessible to proteins, including transcription factors. Haploid germ cells are normally transcriptionally silent from step 10 in mice, thus necessitating the early transcription and storage of mRNAs for their ultimate translation when needed. Of the examples given in S10 Fig, all normally undergo a period of translational delay. Interestingly, four of the most-highly up-regulated genes in meiotic cells, *Tnp1*, *Tnp2*, *Prm1* and *Prm2* are the key transcripts/proteins involved in driving the histone to protamine exchange.

In order to assess the potential for a loss of translational delay in haploid *Henmt1* ^*PIN/PIN*^ cells, which could lead to the relative depletion of the associated mRNAs in round spermatids, testis sections from *Henmt1* ^*PIN/PIN*^ and *Henmt1* ^*WT/WT*^ were immunostained in parallel for TNP1, TNP2, PRM1, PRM2, SPEM1 and GAPDS (S10A–S10F Fig). As expected, all proteins were first observed in elongating spermatids, although the precise step at which they first appeared varied between proteins. Of importance we observed no difference in the timing of first appearance of protein products (translation) between genotypes (*albeit* in the presence of abnormal histology) suggesting that piRNAs are not critically involved in mRNA translational repression in male germ cells.

**Fig 6:**
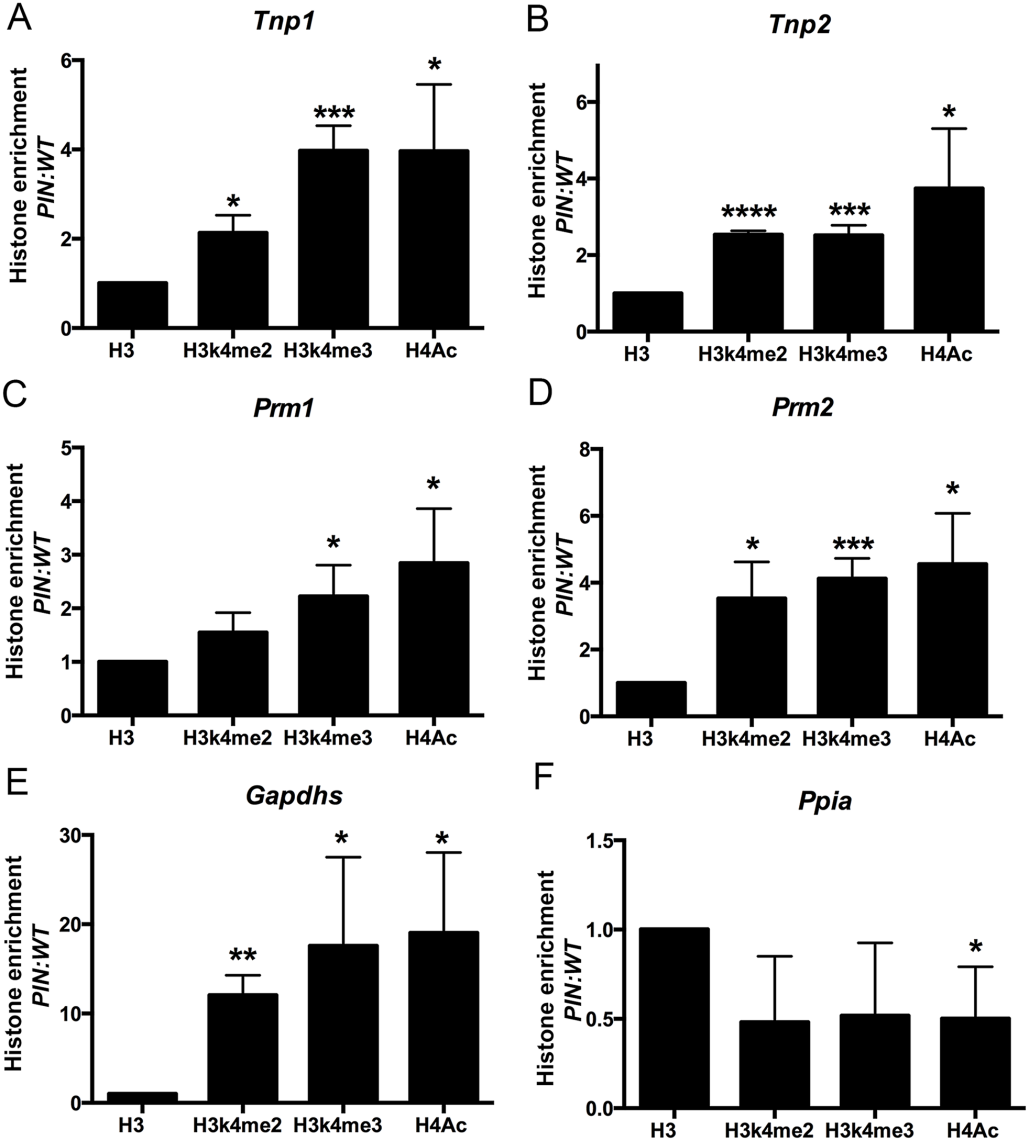
Enrichment of active histone mark at the promoter regions of spermiogenic genes in *Henmt1* ^*PIN/PIN*^ spermatocytes. ChIP and qPCR analyses were performed on *Henmt1* ^*WT/WT*^ and *Henmt1* ^*PIN/PIN*^ spermatocytes (n = 5/genotypes, 3 biological replicates, * p<0.05, **p<0.01, *** p<0.001, **** p<0.0001). qPCR for the promoter regions of (**A**) *Tnp1*, (**B**) *Tnp2*, (**C**) *Prm1*, (**D**) *Prm2*, (**E**) *Gapdhs*, and (**F**) *Ppia* (as a house keeping control). Histone enrichment was normalised to histone H3. The data is presented in the ratio of *Henmt1* ^*PIN/PIN*^ enrichment/ *Henmt1* ^*WT/WT*^ enrichment. A two-tailed unpaired student T-test was performed for statistical analyses.

Thirdly, the elevated expression of haploid germ cell transcripts in *Henmt1* ^*PIN/PIN*^ spermatocytes could be the consequence of changes in chromatin structure in the regulatory regions of these genes in spermatocytes leading to increased access by transcription factors. To assess this possibility, we performed histone ChIP and qPCR in *Henmt1* ^*WT/WT*^ and *Henmt1* ^*PIN/PIN*^ spermatocytes for the active histone marks H3K4me2, H3K4me3 and H4Ac. ChIP was performed using two separate sets of primers designed to the 5’ regulatory regions of *Tnp1*, *Tnp2*, *Prm1*, *Prm2*, *Gapdhs* and *Ppia* as a housekeeping gene whose expression was unchanged between genotypes. Spermatocyte ChIP indicated a significant enrichment of all active histone marks in the 5’ region of all round spermatid genes tested (Fig 6A–6E). As expected, the housekeeping gene *Ppia* was unchanged (Fig 6F). Collectively these data indicate that changes in gene expression in *Henmt1* ^*PIN/PIN*^ spermatocytes were the consequence of a more transcriptionally permissive (active) chromatin state. These data support a role for piRNAs in defining chromatin state and thus, gene expression during male meiosis.

## Discussion

These results clearly show that HENMT1 is required for piRNA 3’ methylation and stability, and ultimately male fertility. In the absence of HENMT1, piRNAs remained unmethylated and were inherently unstable as indicated by their reduced abundance and length, and increased 3’ adenylation and uridylation. piRNA loss resulted in three main effects: the de-repression of at least two classes of TEs in adult germ cells; an apparent shifting forward of the spermatogenesis expression program into meiotic cells associated with more active chromatin state in 5’ regulatory regions; followed by the massive de-regulation of mRNA expression in round spermatids and male infertility as a consequence of extremely abnormal spermatid development.

Our data show that HENMT1 is a critical component of the piRNA metabolic pathway and define that piRNAs are required for both the on-going repression of TEs in meiotic and haploid germ cells, and a normal adult germ cell developmental program. During early germ line development the piRNA pathway, specifically via pre-pachytene piRNAs, is definitively required for the silencing of TEs, including *L1* and *IAP*. This silencing is achieved by a process involving *de novo* DNA methylation by DNMT3a and DNMT3b in partnership with DNMT3L [56–59]. The mechanism by which TE silencing is maintained in later germ cells is largely unknown but as shown here, and in the *Miwi* and *Mili* null mice, requires pachytene piRNAs and is not achieved by DNA methylation in isolation [11,45].

The cell biology of the *Henmt1* ^*PIN/PIN*^ testis suggests an absolutely essential and particularly sensitive role for piRNAs in controlling gene expression in meiotic and haploid germ cells. Surprisingly, spermatogenesis appeared to proceed normally until mid-spermiogenesis in *Henmt1* ^*PIN/PIN*^ mice despite piRNAs and HENMT1 being present throughout spermatogenesis, and the known requirement for piRNAs in TE silencing in prospermatogonia. The reason for this disconnect is not currently known but may be related to transient presence of piRNAs in our model and/or the uncoupling of transcription and translation in late meiotic and haploid germ cells.

In contrast to a recently published report demonstrating a role for piRNAs in mRNA decay in elongating spermatids [48], our analyses provided no evidence of a role for piRNAs in mRNA elimination in spermatocytes. Rather, the majority of piRNAs are sense to mRNA sequences. This data and our ChIP data do, however, support a role for piRNA in promoting a heterochromatic state in meiotic cells. The absence of HENMT1, and the consequent depletion of piRNAs was associated with a significant enrichment of active histone marks in the 5’ regions of numerous ‘round spermatid genes’ in spermatocytes. As a consequence, there was a premature expression of round spermatid transcripts in spermatocytes, and while not tested here, we predict the onset of many secondary effects that ultimately led to the observed complete breakdown of the transcription network in spermatids and their loss from the seminiferous epithelium. As far as we are aware, this is the first evidence of a role for piRNAs in defining chromatin structure in adult male germ cells. It is worth pointing out that data from *Drosophila* wherein Piwi induces piRNA-directed transcriptional silencing has been shown to promote the establishment of H3k9me3 repressive marks and heterochromatin protein 1 (HP1) incorporation into a piRNA target reporter locus [60] are consistent with our wider observations. Future challenges include defining exactly how selective the piRNA-mediated gene silencing is. For example is it restricted to genes involved in cell specific pathways or common pathways shared by many cell types i.e. is it a driver of cell specification? It is also essential to define how piRNAs are modifying chromatin. Are they, for example, directly recruiting histone modifying enzymes onto chromatin?

One of the big questions raised by this study is why was the onset of germ cell abnormalities in *Henmt1* ^*PIN/PIN*^ males later than that seen in *Mili* and *Miwi* knockout mice [12,61]. It is worth restating here that HENMT1 loss of function led to a ~50% reduction in abundance of piRNAs. Those remaining piRNAs were truncated, consistent with destabilisation and more rapid turnover, probably leading to reduced half-life. Minimally truncated piRNAs might be partially functional, and in the context of a tight coupling of transcription and RNA function, as seen in in spermatogonia and early spermatocytes, some piRNAs function may remain. In contrast, the necessity to transcribe then store the bulk of mRNAs, and presumably piRNAs, prior to the histone to protamine transition means that occurs in spermatocytes may lead to a situation wherein piRNAs have a greater opportunity to become degraded prior to their use.

Collectively, this study establishes HENMT1 and piRNA function as absolute requirements for male adult germ cell development and fertility and raises some interesting questions into the causality and treatment of human male infertility. Semen and histological profiles similar to that observed in the *Henmt1* ^*PIN/PIN*^ mouse are regularly observed in infertile men and are referred to as oligoasthenoteratospermia (OAT). The current state-of-the-art treatment for such men is sperm retrieval via testis biopsy followed by intra-cytoplasmic sperm injection (ICSI) [26]. Given the altered chromatin state and massively altered mRNA and piRNA expression profiles observed, and the failure to silence TEs in the remaining ‘sperm-like’ cells in this model, the use of similarly affected gametes from men raises the possibility of trans-generational effects in ICSI-conceived children. While the severity of the sperm abnormalities observed herein could conceivably lead to a failure of early embryonic development, we note a recent study where an apparently mild change in the spermatogenic environment in mice led to an altered small RNA profile in sperm and lifelong trans-generational effects in offspring [62]. Collectively, these studies underscore the importance of small RNAs in adult male germ cells, the necessity for a full explanation of their multiple roles and the influence of environmental impacts on piRNA content for both a male’s fertility and the health of his offspring.

## Materials and Methods

### Mouse strains and mutation mapping

Animal experimentation was approved by the Australian National University and Monash University Animal Experimentation Ethics Committees. ENU mutagenesis was conducted as previously described [63,64]. Point mutant mice were generated on a C57BL/6J background then outbred to CBA. Mouse lines with sterility phenotypes were identified by fertility testing as described previously [63,64]. The causal mutation for male sterility in the Pinhead mouse line was mapped using 5K mouse SNP chip arrays and candidate gene sequencing as previously described [64] which localized the causal mutation to chromosome 3 between nucleotides 108,940,084bp and 108,960,773bp (Ensembl release 73). This region contained 86 genes, 26 of which were expressed in the testis and were thus considered candidates for a causative genetic locus. The full coding sequence of 18 testis-expressed genes was sequenced. A single base pair change was identified in the *Henmt1* gene.

Genotyping of the *Henmt1* ^*PIN*^ allele was performed using the Amplifluor SNP detection system [64]. Primers included a *Henmt1* ^*WT/WT*^ specific sense primer 5’ gaaggtgaccaagttcatgctatcgccatgaacccaagaaggt, a mutant allele sense primer 5’ gaaggtcggagtcaacggattatcgccatgaaccaagaagga and an antisense primer 5’ ccactttaataggggcagtct and Platinum Taq polymerase (Invitrogen).

### Infertility characterization

Male infertility of *Henmt1* ^*PIN/PIN*^ mouse line characterized using the strategies outlined in [65]. Briefly, 8 weeks old *Henmt1* ^*PIN/PIN*^ and *Henmt1* ^*WT/WT*^ males were mated with *Henmt1* ^*WT/WT*^ females (n = 4 / genotype) or *vice versa* over a period of 3 months. *Henmt1* ^*WT/WT*^ and *Henmt1* ^*PIN/PIN*^ females produced litters of similar size i.e. 8.00 ± 1.58 and 6.75 ± 0.85 pups per litter respectively (mean ± S.D, p = 0.5127). We note that by 8 weeks of age mice have established full spermatogenesis and are fertile. *Henmt1* ^*PIN/PIN*^ males however failed to produce any pups despite apparently normal mating behaviour (7.357 vs 0, p = 0.0023). Daily sperm outputs and total epididymal sperm content were determined using the Triton X-100 nuclear solubilization method as described previously (n = 6/genotype) [66]. Sperm motility was determined using a computer assisted sperm analysis system (n = 3/genotype) [67]. Ultrastructure was analyzed using electron microscopy (n = 3/genotype) [68] and caudal epididymal sperm morphology was examined by hematoxylin and eosin staining of air-dried sperm smears.

### Germ cell isolation

Spermatocytes (>90% pachytene) and round spermatids were purified from 28–30 day-old and 10 week-old testes using the Staput method [69]. Spermatocytes had a purity of ≥90% and round spermatids ≥95%.

### TE expression

For *in situ* hybridization a L1 pGEM-T Easy clone was as described previously [70]. *In vitro* transcription was performed to obtain digoxigenin-11-UTP cRNA probes. *In situ* hybridization was performed on adult testis sections as described [71]. LINE-1 protein was detected using a mouse Line-1 (M-300, Santa Cruz) antibody at a concentration of 400ng/ml.

### Antibodies and immunohistochemistry

A synthetic peptide FPTVTLRDADHKFEWSRME encoding amino acids 172–190 (exon 6) of the mouse HENMT1 protein was used to generate a polyclonal goat antibody through Antibodies Australia (Werribee, Australia). The antibody was purified against the immunizing peptide as described previously [72]. This immunogen sequence was contained within all three HENMT1 isoforms. Antibody binding specificity was assured by pre-absorption of the antiserum using a 150-fold molar excess of peptide and through the staining of *Henmt1* ^*PIN/PIN*^ testes where no HENMT1 protein was identified by western blotting (Fig 1F). The γH2AX antibody (Millipore) was used as a marker of double strand break formation. TNP1, TNP2 antibodies were provided by Prof. Stephen Kistler [73]; the SPEM1 antibody was provided by Prof. Wei Yan [53]; and the GAPDS antibody from Prof. Deborah O’Brien [74]. The ABDH5 antibody from Abnova, the PRM1 and PRM2 antibodies were from Briar Patch Biosciences LLC; the CAR2 antibody from Abcam (EPR5195). Immunohistochemistry was performed as previously described [75].

### Western blotting

Proteins were extracted from 10 week-old adult testes (n = 3/genotype) using NP-40 buffer (Fluka). 20μg of protein was separated on a 12% SDS-PAGE gel, proteins transferred to PVDF membranes and then probed using HENMT1 and actin (Sigma Aldrich) antibodies. Bound antibody was detected using donkey anti-goat IgG HRP (Dako) secondary antibody and donkey anti-rabbit IgG HRP respectively and detected with enhanced chemiluminescence (ECL Plus) detection kit (Thermo Scientific).

### Quantitative PCR

Total RNA was extracted using Trizol reagent (Invitrogen) and converted into cDNA using SuperScript III (Invitrogen). Taqman Assays were purchased (Life Technologies) and used to quantitate the expression of *Henmt1* isoform 1 and 2 (Mm00659237_m1), *Ppi* (Mm02342429_g1), *Tnp1* (Mm04207755_g1), *Tnp2* (Mm00726979_s1), *Prm1* (Mm01342731_g1), *Prm2* (Mm03048199_m1), *Gapds* (Mm00484668_m1), *Spem1* (Mm01250806_g1), and *Car2* (Mm00501576_m1). *L1_Md_A*, *L1_Md_TF14* and *Henmt1* transcript 3 specific assays were custom made and primers and probe sequence are listed in S3 Table. The expression of *IAP_LTR* and *IAP_GAG* was detected using Brilliant Fast SYBR Green qPCR master mix (Stratagene). All PCRs were performed in the Agilent Mx3000P qPCR system: 95°C, 2 min; 95°C, 30 sec; 60°C 30 sec, 72°C 45 sec, repeat 40 cycles. *Ppia* expression was used as a reference for gene expression analysis from Taqman assays while *18S* expression was used as reference for analysis from SYBR Green qPCR. Differential expression was analyzed using the 2^ΔΔCT^ method [76]. A student’s T-test was performed to compare the means of two populations via Graphpad Prism 5.0. P values <0.05 was considered statistical significance.

### β-elimination assay and small RNA quantitation

To determine the presence of 2’-O-methylation at the 3’end of piRNA, 50 μg of total RNA was purified from 10 week-old testes then treated with NaIO_4_ for 1 hour. After treatment RNA was precipitated at -20°C overnight then re-suspended with borate buffer (pH 9.5) for β-elimination treatment at 45°C 90 min [29,77]. RNA was purified through a G25 column (GE Healthcare) to remove salts then loaded on 15% TBE Urea gels (Life technologies) and blotted onto a membrane via the xCell Blot Module (Invitrogen) according to the manufacturer’s protocol. Blots were prehybridized with the Dig easy Hyb buffer (Roche) for 2 hours and hybridized overnight at 42°C with a γP^32^-labelled piRNA1 oligo probe [47]. Membranes were washed with increasing stringency, 3XSSC/25mM NaH_2_PO_4_/5% SDS and 1xSSC/1%SDS, then exposed to phospho-imager screens prior to scanning on a Typhoon Trio (GE Healthcare).

For the quantitation of piRNA with 30 day old testes, total RNAs were isolated and size separated on 15% TBE urea gels as described above, then the relative piRNA content calculated between wild type and *Henmt1* ^*PIN/PIN*^ samples using densitometry via the Image Lab image acquisition and analysis software (Bio-Rad). In brief this involved piRNA band (~30nt) was normalized to loading control 5s rRNA and 5.8srRNA bands.

### Histone Chromatin immune precipitation (ChIP) and qPCR

Spermatocytes and round spermatids were purified from *Henmt1* ^*WT/WT*^ and *Henmt1* ^*PIN/PIN*^ animals using the Staput method (n = 5 mice per replicate by three biological replicates). 5x10^5^ cells were used for each pull down. Cells proteins and DNA were cross-linked with 1% formaldehyde then quenched with 0.125M glycine as published previously [78]. Cells were washed and lyzed in ice-cold lysis buffer (10mM Tris, 10MM NaCl, 0.2% NP-40, protease inhibitors) and sonicated at 4°C for 30 minutes. ChIP was performed with antibodies against the active histone marks H3K4me2 (Millipore), H3K4me3 (Millipore) and H4Ac (Abcam) and control histone H3 (Abcam) and captured with protein A Sepharose. DNA-protein complex were washed with IP buffer I (20mM Tris-HCl, 2mM EDTA, 50mM NaCl, 0.1% SDS and 1% Triton X), twice with buffer II (10mM Tris, 1mM EDTA, 0.25 M LiCl, 1% NP-40, 1% Na-deoxycholate) then eluted in elution buffer (100mM NaHCO_3_ and 1%SDS). Eluted fractions was treated with 2μl of RNAse A (10 mg/ml) and 3μl of proteinase K (20 mg/ml) at 65°C overnight. DNA was then extracted using phenol/chloroform extraction.

Two sets of PCR primers were designed at the promoter region of *Prm1*, *Prm2*, *Tnp1*, *Tnp2*, *Gapdhs* and control gene *Ppia*. Specificity of each primer set was confirmed by sequencing of the PCR products. qPCR with Brilliant Fast SYBR Green QPCR Master Mix (Stratagene) in the Agilent Mx3000P qPCR system: 95°C, 1 min; 95°C, 10 sec; 60°C, 10 sec; 72°C, 10 sec, for 40 cycles, 95°C, 10 sec; 65°C, 1 min; 97°C, 1 sec. Primer sequences were as listed in S4 Table.

### RNA sequencing

Two biological replicates for *Henmt1* ^*WT/WT*^ spermatocytes and three replicates for other genotypes, each consisting of germ cells isolated from N = 2–3 animals were used for sequencing. 1μg of RNA was used for library construction.

For small RNA sequencing, total RNA was qualified using an Agilent BioAnalyzer and 1 μg was used for library construction with Illumina's TruSeq Small RNA sample preparation kit, according to the manufacturer's instructions (TruSeq SmallRNA SamplePrep Guide RevE). Size selection of the small RNA libraries following ligation of the adapters was done using a Pippen Prep 3% gel with an elution size range of 122–182 base pairs accounting for both miRNAs (19–22 nt) and piRNAs (26–31 nt) plus adapter sequences. Small RNA library size and yield was validated with an Agilent BioAnalyzer. The small RNA libraries were sequenced in an Illumina HiSeq 2000 sequencer (50 bp single-end reads) at the Australian Cancer Research Foundation Cancer Genomics Facility (Adelaide). The TruSeq adapter 'TGGAATTCTCGGGTGCCAAGGAACTCCAGTCAC' was removed from the 3' end of reads using Cutadapt v1.3, requiring a minimum overlap of 5 bases, allowing 20% error rate in bases matched, and discarding sequences of less than 18 bases. No quality trimming was performed as it would cause a 3’ truncation bias. Resulting reads were mapped to the mouse reference genome (mm10) using BWA v0.7.3a-r367, allowing at most 3 alignments.

For mRNA expression analysis total RNA was qualified using an Agilent BioAnalyzer and 1 μg was used for polyA selection and library construction with Illumina's TruSeq RNA sample preparation kit according to the manufacturer's instructions (TruSeq RNA SamplePrep v2 Guide RevD). The mRNA library size and yield was validated with the Agilent BioAnalyzer. The mRNA libraries were sequenced in an Illumina HiSeq 2000 sequencer (2 x 100 bp paired-end reads) at the Australian Cancer Research Foundation Cancer Genomics Facility. Reads were mapped against the mm10 reference genome with Tophat2 v2.0.9 [79] with the—GTF option (assign isoforms using reference transcripts), using the Illumina iGenomes UCSC mm10 gene annotations. Differential expression of mouse RefSeq genes (as of July of 2013 from UCSC) were analyzed using edgeR. Functional annotations of up-regulated or down-regulated genes were performed using DAVID Functional Annotation Tool (http://david.abcc.ncifcrf.gov/).

### Heatmap and volcano plot of differentially expressed genes

Within spermatocytes 256 genes were significantly up-regulated and 12 significantly down-regulated genes as identified using edgeR (FDR < 0.05). The fold change of these genes between *Henmt1* ^*PIN/PIN*^ and *Henmt1* ^*WT/WT*^ in spermatocytes and round spermatids were calculated with the edgeR package and plotted as a heatmap. With respect to the volcano plot, the fold change of all genes with detectable expression (CPM/counts per million > 1 in at least 2 RNA-seq samples) between *Henmt1* ^*PIN/PIN*^ and *Henmt1* ^*WT/WT*^ in spermatocytes and round spermatids were calculated with the edgeR package. The significantly differentially expressed (up or down-regulated) genes were determined based on adjusted P value (FDR) < 0.05 according to edgeR.

### piRNA size distribution

Read lengths were tallied for sequences matching our piRNA length and sequence criteria. Length counts were expressed as a percentage of total piRNA reads in the sample. Replicates were grouped together by condition (*Henmt1* ^*WT/WT*^ or *Henmt1* ^*PIN/PIN*^) and shown with a boxplot. A dashed line was plotted between the mean of the replicates. Separate graphs were produced for spermatocytes and round spermatids.

### piRNA 3’ end analysis

We examined that last 3' base of each aligned read identified as a piRNA. If the reference base for that position was not 'T' (and thus it was possible to determine whether a base had been modified), we counted whether the read was 'T' (indicating uridylation) or not. Adenylation was measured similarly, checking for ‘A’.

### piRNA classification and mapping

Small RNA reads were annotated based on overlapping with (at least 1bp) genomic coordinates of known piRNA clusters (http://www.uni-mainz.de/FB/Biologie/Anthropologie/492_ENG_HTML.php), repeats, protein-coding RNAs and non-coding RNAs in mouse RefSeq (as of July of 2013 from UCSC). Reads that had matches with more than one datasets were classified exclusively in the order of piRNA > repeat > exon of protein-coding RNA > exon of non-coding RNA > intron. Reads mapped to the antisense strand of above datasets were further annotated as antisense small RNAs. All the other reads were classified as intergenic type. Reads mapped to piRNA clusters were annotated as candidate piRNAs and further classified based on above known datasets with the same procedure. Candidate piRNAs mapped to protein-coding RNAs were further annotated as 5’ UTR-type, CDS-type and 3’ UTR-type based on the overlaps with corresponding regions of mRNAs.

### piRNA expression analysis

Global piRNA expression analysis tools were used to produce a description of piRNA expression level by genomic location and piRNA length. The complete code is available from the repository at https://github.com/henmt/2015. A number of parameterized options are provided that allow tailoring of the analysis. For example, piRNA type filtering; mapping quality filtering; piRNA deduplication by denesting reads; genomic bin size adjustment. The approach we used was that BAM alignments were read and filtered on sequence and mapping quality. Alignments that passed these filters were then filtered optionally on piRNA 1° or 2° status. Alignments were then counted, recording their length and position and unique 5' ends were counted as a proxy for piRNA family since piRNAs appear to truncate primarily from the 3' end. If denesting was requested, 5' ends of alignments were only considered if the alignment was not fully encompassed by another alignment. Denesting was performed by keeping a record of all unique alignment intervals in an interval tree and then after reading all alignments, unique 5' ends were identified as intervals with no containing interval.

A differential piRNA expression analysis tool was used to produce a description of piRNA expression level differences by genomic location and piRNA length. The complete code is available from the repository at https://github.com/henmt/2015. A number of parameterized options are provided that allow tailoring of the analysis such as: piRNA type filtering; feature overlap filtering; mapping quality filtering; piRNA deduplication by denesting reads; genomic bin size adjustment. The approach we took was that two BAM alignments were read and filtered on sequence and mapping quality. Alignments that passed these filters were then filtered optionally on piRNA 1° or 2° status. Alignments that passed these filters were then assessed for overlap with an optionally provided set of features from a GFF file and a set of feature classes to compare against. If these were given only alignments that overlap the specified classes of features were included in the analysis. Alignments were then counted, recording their length and position and unique 5' ends are counted as a proxy for piRNA family since piRNAs appear to truncate primarily from the 3' end. If denesting was requested, 5' ends of alignments were only considered if the alignment was not fully encompassed by another alignment. The difference between the two inputs' bin and length tallies were then calculated and stored. Unique 5' end counts were kept as individual data from each sample. Assumptions—we were interested in the source of the piRNA rather than the targets of the piRNA in this instance. We assumed the source would match the read better than the target and that only a small number of mismatching reads would be reported (and further that if they mismatched too much they would be culled by percentage id filtering). In the case of repeats, some proportion of the piRNA source loci would have been surrounded by enough target-like sequence that it would be recognized by RepeatMasker and so would have been annotated. Denesting was performed by keeping a record of all unique alignment intervals in an interval tree and then after reading all alignments, unique 5' ends were identified as intervals with no containing interval.

In order to calculate the degree of 5’ and 3’ end truncation we used overlap-end generates a csv of values and associated plot describing truncation of piRNA alignments from two non-overlapping size pools. The complete code is available from the repository https://github.com/henmt/2015. A number of parameterized options are provided that allow tailoring of the analysis: pool alignment lengths (short and long); piRNA type filtering; mapping quality filtering; long pool piRNA deduplication by denesting reads; and short alignment containment. The approach we took was that read alignments from one or two BAM files—if two files were specified one was nominally wild type and the other nominally mutant, long alignments (default 28-32bp) were taken from the wild type data and short alignments (default 23-27bp) were taken from the mutant data. These two data sets were then compared as follows. One instance of each unique alignment was kept from the longer pool, optionally denesting the unique long pool alignments. The initial uniqueness test was done by start/length identity (map assignment). For each alignment from the shorter pool find all long alignments that overlapped their map location by at least one base in the non-contained case or all long alignments that completely overlap it in the contained case. We recorded the relative end positions between the short alignment and the found long alignments (transformed so that lengthening would be a positive distance and shortening would be a negative distance). Denesting was performed by keeping a record of all unique alignment intervals in an interval tree and then after reading all alignments, canonical long piRNA alignments were identified as intervals with no containing interval. Containment of short reads was intended to reduce the linear scoring behaviour in response to long pool read truncations; a long pool with a number of truncated forms that overlap with short alignments would otherwise multiply out the counts of truncations. It was also intended to abolish a 'negative' truncation effect where short alignments were determined to be negatively truncated from a long alignment overlaps, but did not completely contain the short alignment. When contain was requested as an option (the default), long alignments were only considered for comparison to a short alignment when they completely overlapped the short alignment and were longer than the short alignment.

### Methylation Sensitive Southern Blot

DNA purification: cell pellets of ~one million pachytene spermatocytes (PS) or round spermatids (RS) were incubated in 0.3 mL of lysis/digest buffer [50 mM Tris-HCl pH 8.0, 100 mM NaCl, 100 mM EDTA, 1% (w/v) SDS, 0.3 mg/mL proteinase K] (55°C, 4 h). 0.1 mL of 10M ammonium acetate was added and the tube vortexed to bring the SDS out of solution. The SDS was pelleted (4°C, 14,000 rpm, 12 min) and the supernatant collected. The supernatant was then extracted with phenol/chloroform (0.2 mL, 1×) then with chloroform (0.3 mL, 2×). An equal volume of isopropanol was added, the tube vortexed, and stored at -30°C overnight. Genomic DNA was then pelleted (4°C, 14,000 rpm, 12 min), and the supernatant removed. The pellet was washed twice with 70% (v/v) ethanol, dried, and resuspended in 40 μL of TE. DNA recovery was ~2.5 μg for PS, and ~4 μg for RS.

Restriction digests and transfer to nylon filters: 1.0 μg of each sample was digested with 20 U of HpaII or 30 U of MspI in 40 μL for 8 h (NEB; enzymes and CutSmart buffer), then 10 μL of 6× Ficoll loading buffer added. For the LINE1 A family (L1Af) blot, 20 μL/well was loaded in a 1.5% agarose gel and electrophoresed. DNA in the gel was then denatured, neutralized, and capillary-transferred with 10× SSC to GeneScreen Plus (NEN Life Science Products) membrane according the manufacturer’s instructions. DNA was cross-linked to the membrane using UV light, or by baking. For the ERVII (intracisternal A-particle) and mitochondrial DNA blot, details are as above except that 0.8% gels were used, and that DNA was depurinated performed before the denaturation step.

Probes: The LIAf probe was synthesized (GenScript), corresponding to a contig of bases 1248–1403 and 1539–1694 of GenBank accession no. M13002, and recognizes sequences as previously described (Bourc’his and Bestor 2004, PMID:15318244). The total sequence is: GAATTCGGGC TACCTTGACA GCAGAGTCTT GCCCAACACC CGCAAGGGCC CACACGGGAC TCCCCACGGG ACCCTAAGAC CTCTGGTGAG TGGAACACAG CGCCTACCCC AATCCAATCG CGTGGAACTT GAGACTGCGG TACATAGGGA AGCAGGCTAC CCACTTCTGC CAGGAGTCTG GTTCGAACAC CAGATATCTG GGTACCTGCC TTGCAAGAAG AGAGCTTGCC TGCAGAGAAT ACTCTGCCCA CTGAAACTAA GGAGAGTGCT ACCCTCCAGG TCTGCTCATA GAGGCTAACA GAGTCACCTG AAGAACAAGA ATTC (appended *EcoR*I sites are underlined). The ERVII and mitochondrial DNA probes used were pERV2-ORF1 and pMIT1 (Ip et al. 2012, PMID:22969435). Radioactive ^32^P probes were made by random-priming (Megaprime DNA Labeling system, GE Healthcare).

Hybridization and washing: Hybridization was carried out in 5× SSPE, 5× Denhardt’s solution, and 1% (w/v) SDS (65°C, overnight). The L1Af probing was washed with 0.8× SSC (65°C, 10 min; 3×), while the mitochondrial and ERVII probings were washed with 0.8× SSC (65°C, 10 min; 1×), then 0.2× SSC (65°C, 10 min; 2×). Filters were scanned with a Typhoon PhosphorImager (GE Healthcare). Control animals are *Piwil2* heterozygous mice and *Piwil2* Knock-out mice which L1 are hypomethylated.

## Supporting Information

S1 Table Sequence based criteria of piRNAs. (DOCX)

S2 Table A. Size distribution of MILI-loaded piRNAs (S4C Fig). B. Size distribution of MIWI-loaded piRNAs (S4D Fig).
(DOCX)

S3 Table Custom made Taqman probes and primers used for qPCR. (DOCX)

S4 Table Primers for ChIP-qPCR. (DOCX)

S1 Dataset **Tab 1,** Genes that were expressed in pachytene spermatocytes and round spermatids. **Tab 2,** Genes that were significant up-regulated in pachytene and down-regulated in round spermatids. **Tab 3,** piRNA expression level in spermatocytes up-regulated genes. **Tab 4, 5**, Gene Ontology (GO) term analysis of genes under-going different type expression changes between meiotic and haploid cells.
(XLSX)

S1 Fig *Henmt1* mRNA is highly expressed in the testis. **(A)** Quantitative real-time PCR analysis for *Henmt1* isoform 3 in 8 week old *Henmt1* ^*PIN/PIN*^ *and Henmt1* ^*WT/WT*^ testis. Values are relative to wild type testis (n = 3 / genotype +/- SD) (p*<0.05, ** p<0.01, mean ± SD). Two-tailed unpaired student T test was performed for statistical analyses. **(B)** qPCR analysis for *Henmt1* mRNA in tissues (all isoforms). Values are relative to wild type testis (n = 3 / genotype +/- SD). **(C)** *Henmt1* mRNA expression during the establishment of the first wave of spermatogenesis relative to day 0 expression (all isoforms, n = 3/ genotype).
(TIF)

S2 Fig Electron microscopic abnormalities in *Henmt1* ^*PIN/PIN*^ in spermatids. Electron microscopy demonstrating the presence of mitochondria in (**A)** *Henmt1* ^*WT/WT*^ and (**B)** *Henmt1* ^*PIN/PIN*^ elongating spermatids. Red thin arrows represent normal mitochondria distribution. Red thick arrows represents abnormal mitochondria clustering. **(C)** Step 16 *Henmt1* ^*WT/WT*^ spermatids showing a well-organized mitochondrial sheath (red arrow) around the axoneme of the tail (yellow arrow). (**D)** Step 16 *Henmt1* ^*PIN/PIN*^ spermatids with aggregated mitochondria and a poorly organized mitochondrial sheath around the axoneme (yellow arrow). **(E)** A stage IX tubule. In *Henmt1* ^*WT/WT*^ (left hand side for the remainder of panels), step 9 spermatids containing normal machettes structures composed of a perinuclear ring (yellow arrow) adjacent to the caudal end of developing acrosome (ac) and an associated fringe of microtubules (mt). Step 9 *Henmt1* ^*PIN/PIN*^ spermatids (right hand side in all panels) displayed abnormal manchette development. The perinuclear ring was orientated towards the plasma membrane. **(F)** A stage I *Henmt1* ^*WT/WT*^ tubule containing step 13 spermatids showing a normal machette (red box) and condensed spermatids (arrow). *Henmt1* ^*PIN/PIN*^ step 13 spermatids frequently contained ectopically placed machettes (red box) and heterogenous levels of chromatin condensation (arrows). **(G)** A *Henmt1* ^*WT/WT*^ step 5 (stage V) spermatids containing normal chromatoid bodies composed of filaments that formed an irregular network of electron dense cords (arrowhead). Step 5 *Henmt1* ^*PIN/PIN*^ spermatids contained similar, normal, intact chromatoid bodies (arrowhead). **(H)** *Henmt1* ^*WT/WT*^ and *Henmt1* ^*PIN/PIN*^ pachytene spermatocytes contained comparable inter-mitochondrial cement structures (stage VIII) (white arrow). Cartoons represent the germ cell types present within the seminiferous epithelium at the stage being analyzed.
(TIF)

S3 Fig Total piRNA content in *Henmt1* ^*WT/WT*^ testes. The percentage piRNAs classification in *Henmt1* ^*WT/WT*^ spermatocytes (**A**) and round spermatids (**B**) from deep sequencing analyses.
(TIF)

S4 Fig Both MILI and MIWI-associated piRNAs were truncated in *Henmt1* ^*PIN/PIN*^ germ cells. Boxplots showing the percentage of size selected reads defined as MILI (**A**) and MIWI-loaded (**B**) piRNAs in purified spermatocytes and round spermatids by overlapping with coordinates from HITS-CLIP data derived from whole mouse testis [33]. (**C-D**) The size distribution of MILI-loaded (C) and MIWI-loaded piRNAs (D), as defined by HITS-CLIP data [33], from *Henmt1* ^*WT/WT*^ and *Henmt1* ^*PIN/PIN*^ spermatocytes and round spermatids. These panels demonstrate the shifting of the length distribution of piRNAs to the left in *Henmt1* ^*PIN/PIN*^ compared to *Henmt1* ^*WT/WT*^ that results from end truncation. Please see S2 Table for the actual data used to plot the figure.
(TIF)

S5 Fig Global piRNA expression. (**A**) Global expression of primary piRNA species in spermatocytes was significantly localized to a small number of well-defined loci. A representation of mapping frequency of cutadapt-trimmed primary piRNA sequences to 10Mbp bins of the mouse genome. The heat map shows piRNA mapping counts per base pair with each bin stratified by piRNA length from 20 bp (inner edge) to 35 bp (outer edge) inclusive. The outer trace track shows the summed piRNA mapping counts per base for long (28-32bp inclusive; blue) and short (23-27bp inclusive; red) piRNA species. The inner trace track shows the number of distinct 5' ends mapped to each bin location. (**B)** piRNAs in *Henmt1* ^*PIN/PIN*^ germ cells were shortened. Differential expression analysis of primary piRNA species in spermatocytes showed an overall reduction in *Henmt1* ^*PIN/PIN*^ cells of long piRNA species and an increase of short piRNA species. A representation of differential mapping frequency of all cutadapt-trimmed primary piRNA sequences mapped to 10Mbp bins of the mouse genome. The heat map shows differential piRNA mapping counts (mutant vs wild type) per base pair in each bin stratified by piRNA length from 20 bp (inner edge) to 35 bp (outer edge) inclusive. There is a clear deficit of longer piRNAs and clear excess of shorter piRNAs in mutant spermatocytes as evidenced by this result. The outer trace track shows the summed change in piRNA mapping counts per base for long (28-32bp inclusive; blue) and short (23-27bp inclusive; red) piRNA species. The inner trace track shows the number of distinct 5' ends mapped to each bin for wild type (green) and mutant (magenta). A description of the analysis strategy is provided in the Materials and Methods. (**C)** The expression of TE-directed piRNAs in *Henmt1* mutant spermatocytes. Differential expression analysis of L1-targeted primary piRNA species (piRNAs with sequence similarity to L1 elements) in spermatocytes showed an increase in *Henmt1* ^*PIN/PIN*^ cells of long piRNA species at the chromosome 17 piRNA cluster. A representation of differential mapping frequency of LINE-specific cutadapt-trimmed primary piRNA sequences mapped to 10Mbp bins of the mouse genome. The heat map shows differential piRNA mapping counts per base pair each bin stratified by piRNA length from 20 bp (inner edge) to 35 bp (outer edge) inclusive. The outer trace track shows the summed change in piRNA mapping counts per base for long (28-32bp inclusive; blue) and short (23-27bp inclusive; red) piRNA species. The inner trace track shows the number of distinct 5' ends mapped to each bin for wild type (green) and mutant (magenta). A description of the analysis strategy is provided in the Materials and Methods.
(TIF)

S6 Fig piRNA truncation occurs differentially at the 5' and 3' ends and is impacted by *Henmt1* genotype. Quantification of truncation of primary piRNA ends from spermatocytes represented by end-length change where negative length change signifies truncation. **(A)** Proportions of primary piRNA populations from wild type spermatocyte tissue showing 5' (blue) and 3' (red) end truncation. There was no significant increase in the truncation of piRNA at the 5' end compared to the 3' end. Longer truncations (four bases or more) are significantly more frequent at the 3' end than at the 5' end. Results for secondary piRNA and samples taken from round spermatids showed qualitatively similar patterns. A description of the analysis strategy is provided in the Materials and Methods. The 95% confidence interval for the extent of 3' end truncation compared to 5' end truncation: wild type spermatocyte 1°, 1.866477–1.872112; wild type spermatid 1°, 1.904809–1.913252; wild type spermatocyte 2°, 2.128158 2.139266; wild type spermatid 2°, 2.203954 2.217913; mutant spermatocyte 1°, 2.024495–2.028101; mutant spermatids 1°, 2.014840–2.019387; mutant spermatocyte 2°, 2.413812–2.421914; mutant spermatids 2°, 2.371488–2.379345). **(B)** Proportions of primary piRNA populations from wild type (blue) and mutant (red) spermatocytes showing 3' end truncation. Small (five or fewer bases) length changes were elevated in wild type compared to mutant while larger length changes were elevated in mutant. The 95% confidence intervals for change in 3' end truncation in *Henmt* ^*PIN/PIN*^: spermatocyte 1°, 0.2749190–0.2798546; spermatid 1°, 0.2761497–0.2832534; spermatocyte 2°, 0.2835850–0.2939058; spermatid 2°, 0.2756250–0.2877403).
(TIF)

S7 Fig The presence of ping-pong derived piRNA in adult germ cell types. Stringent ping-pong cycle derived piRNAs were defined by the following criteria: 1) size from 23-32nt; 2) 1^st^ base of primary (1°-piRNA) piRNA is “U” or 10^th^ base of secondary (2°-piRNA) piRNA is “A”; 3) primary (1°-piRNA) piRNA should have 10 nt overlap at the 5’ end with its corresponding secondary (2°-piRNA) piRNAs at the 5’ end; 4) primary (1°-piRNA) piRNA should be mapped to repeats or protein-coding RNAs in sense and secondary (2°-piRNA) piRNA should be mapped to repeats or protein-coding RNAs in antisense. **(A)** Proportion of ping-pong derived piRNAs in all piRNAs. **(B)** Proportion of primary (1°-piRNA) or secondary (2°-piRNA) ping-pong piRNAs mapped to all repeats in all piRNAs. (**C)** The proportion of primary (1°-piRNA) or secondary (2°-piRNA) ping-pong piRNAs mapped to all protein-coding genes in all piRNAs.
(TIF)

S8 Fig Ping-pong cycles measured by ping-pong z score. (**A**) Z scores of the different overlapping lengths of piRNA pairs from 5’ end showed biased 10 nt overlap, which is a signature of ping-pong piRNA amplification. (**B**) Decreased ping-pong z scores were observed in both *Henmt1* ^*PIN/PIN*^ spermatocytes and round spermatids compared to *Henmt1* ^*WT/WT*^.
(TIF)

S9 Fig HENMT1 dysfunction was associated with increased *L1* expression but not with changes in global DNA methylation. **(A)** Transcriptome analysis of *Henmt1* ^*WT/WT*^ and *Henmt1* ^*PIN/PIN*^ spermatocytes and round spermatids demonstrated increased *L1_A* and *L1_TF14* expression in the *Henmt1* ^*PIN/PIN*^ spermatocytes (PS) and round spermatids (RS). (**B-C)** gDNA was digested with methylation sensitive enzymes (HpaII) and methylation insensitive enzyme (MspI) * gDNA digested with MspI, (**B**) Southern blot was hybridized with L1 probe (**C**) with an ERVII probe. Mito probe is used for loading control. The arrow indicates the presence of methylation sensitive restriction products in the control testis.
(TIF)

S10 Fig Translational delay was not affected by HENMT1 dysfunction. Immunostaining for of key spermatid proteins in *Henmt1* ^*WT/WT*^ (left panel) and *Henmt1* ^*PIN/PIN*^ (right panel) in full spermatogenesis. **(A)** PRM1, (**B)** PRM2, (**C)** TNP1, (**D)** TNP2, (**E)** SPEM1 and (**F)** GAPDHS. Scale bar = 100μm.
(TIF)
