# Correction: HENMT1 and piRNA Stability Are Required for Adult Male Germ Cell Transposon Repression and to Define the Spermatogenic Program in the Mouse

**DOI:** 10.1371/journal.pgen.1005782

**Published:** 2015-12-29

**Authors:** Shu Ly Lim, Zhi Peng Qu, R. Daniel Kortschak, David M. Lawrence, Joel Geoghegan, Anna-Lena Hempfling, Martin Bergmann, Christopher C. Goodnow, Christopher J. Ormandy, Lee Wong, Jeff Mann, Hamish S. Scott, Duangporn Jamsai, David L. Adelson, Moira K. O’Bryan

There is an error in the designations for equal authorship contributions. The last author, Moira O’Bryan, should not have been attributed equal contributions with authors 2, 3 and 4. Authors 2, 3, and 4, Zhi Peng Qu, R. Daniel Kortschak and David M. Lawrence contributed equally to this work.
