# Supplementary material for: HENMT1 and piRNA Stability Are Required for Adult Male Germ Cell Transposon Repression and to Define the Spermatogenic Program in the Mouse
Source: PLoS Genet. 2015 Oct 23;11(10):e1005620. doi: 10.1371/journal.pgen.1005620 (PMC4619860; doi:10.1371/journal.pgen.1005620)
Supplement: S1 Table — (DOCX) [file pgen.1005620.s001.docx]

| **Genotype** | | **Sample type** | | **1° piRNA counts (%)** | | **2° piRNA counts (%)** | | **1°:2°** |
| --- | --- | --- | --- | --- | --- | --- | --- | --- |
| *Henmt1^WT/WT^* | | Spermatocytes | | 10752104 (90.31%) | | 1152847 (9.69%) | | 9.319 |
| *Henmt1^WT/WT^* | | Spermatocytes | | 21961940 (90.16%) | | 2396147 (9.84%) | | 9.162 |
| *Henmt1^WT/WT^* | | Round spermatids | | 3006853  (89.20%) | | 13113125 (10.80%) | | 8.260 |
| *Henmt1^WT/WT^* | | Round spermatids | | 2084817 (89.89%) | | 11278827 (11.11%) | | 8.090 |
| *Henmt1^WT/WT^* | | Round spermatids | | 15764840 (89.46%) | | 1761983 (10.54%) | | 8.488 |
| *Henmt1^PIN/PIN^* | | Spermatocytes | | 15764840 (90.92%) | | 1574554 (9.08%) | | 10.013 |
| *Henmt1^PIN/PIN^* | | Spermatocytes | | 17307687 (90.76%) | | 1763766 (9.24%) | | 9.822 |
| *Henmt1^PIN/PIN^* | | Spermatocytes | | 17033659 (91.36%) | | 1611030 (8.64%) | | 10.574 |
| *Henmt1^PIN/PIN^* | | Round spermatids | | 2977804 (90.95%) | | 17490799 (9.05%) | | 10.050 |
| *Henmt1^PIN/PIN^* | | Round spermatids | | 2176999 (90.47%) | | 11995390 (9.53%) | | 9.493 |
| *Henmt1^PIN/PIN^* | | Round spermatids | | 2509178 (90.53%) | | 14477510 (9.47%) | | 9.560 |
| **Average of each cell type per genotype** | | | | | | | | |
| *Henmt1^WT/WT^* | Spermatocytes | | 16357022 (90.24%) | | 1774497 (9.76%) | | 9.246 | |
| *Henmt1^PIN/PIN^* | Spermatocytes | | 16702062 (91.01%) | | 1649783 (8.99%) | | 10.123 | |
| *Henmt1^WT/WT^* | Round spermatids | | 11542129 (89.20%) | | 1398494 (10.80%) | | 8.259 | |
| *Henmt1^PIN/PIN^* | Round spermatids | | 14654566 (90.44%) | | 1550625 (9.56%) | | 9.460 | |
