# Supplementary material for: HENMT1 and piRNA Stability Are Required for Adult Male Germ Cell Transposon Repression and to Define the Spermatogenic Program in the Mouse
Source: PLoS Genet. 2015 Oct 23;11(10):e1005620. doi: 10.1371/journal.pgen.1005620 (PMC4619860; doi:10.1371/journal.pgen.1005620)
Supplement: S2 Table — A. Size distribution of MILI-loaded piRNAs (S4C Fig). B. Size distribution of MIWI-loaded piRNAs (S4D Fig). (DOCX) [file pgen.1005620.s002.docx]

**Table S2A:** size distribution of MILI-loaded piRNAs (Fig S4 C)

|  | Spermatocytes | | | | Round spermatids | | | |
| --- | --- | --- | --- | --- | --- | --- | --- | --- |
|  | *WT/WT* | | *PIN/PIN* | | *WT/WT* | | *PIN/PIN* | |
| Length | %* | se** | % | se | % | se | % | se |
| 23 | 1.02195 | 0.036822 | 1.817131 | 0.157726 | 1.003575 | 0.072784 | 1.85783 | 0.139798 |
| 24 | 1.545261 | 0.04116 | 3.364672 | 0.218084 | 1.124767 | 0.085301 | 2.983951 | 0.154467 |
| 25 | 2.692187 | 0.184836 | 6.029643 | 0.324844 | 1.543637 | 0.109159 | 4.754082 | 0.175857 |
| 26 | 5.401938 | 0.329721 | 8.598015 | 0.352203 | 3.045254 | 0.15892 | 6.489911 | 0.185894 |
| 27 | 6.903919 | 0.360701 | 7.837768 | 0.208899 | 3.980273 | 0.153098 | 6.729363 | 0.155343 |
| 28 | 8.578739 | 0.416941 | 8.302698 | 0.189531 | 6.689708 | 0.094873 | 9.351567 | 0.158863 |
| 29 | 17.37697 | 0.0343 | 16.69625 | 0.13866 | 18.90711 | 0.253457 | 21.48067 | 0.511333 |
| 30 | 28.89566 | 0.62855 | 24.61195 | 0.547016 | 32.68175 | 0.9332 | 25.72421 | 0.392006 |
| 31 | 19.79867 | 0.613815 | 16.70451 | 0.531102 | 21.53664 | 0.830966 | 14.85376 | 0.105489 |
| 32 | 7.784703 | 0.162116 | 6.037362 | 0.27091 | 9.487274 | 0.926962 | 5.774658 | 0.122788 |

*, percentage; **, standard error

**Table S2B:** size distribution of MIWI-loaded piRNAs (Fig S4 D)

|  | Spermatocytes | | | | Round spermatids | | | |
| --- | --- | --- | --- | --- | --- | --- | --- | --- |
|  | *WT/WT* | | *PIN/PIN* | | *WT/WT* | | *PIN/PIN* | |
| Length | %* | se** | % | se | % | se | % | se |
| 23 | 0.964021 | 0.034903 | 1.737398 | 0.151238 | 0.950245 | 0.06967 | 1.777863 | 0.143198 |
| 24 | 1.498914 | 0.070716 | 3.334863 | 0.22141 | 1.084554 | 0.101101 | 3.050142 | 0.201261 |
| 25 | 2.795945 | 0.219195 | 6.219408 | 0.329057 | 1.62927 | 0.146201 | 5.186547 | 0.30177 |
| 26 | 5.843489 | 0.359367 | 9.13406 | 0.351356 | 3.312657 | 0.22404 | 6.870228 | 0.330651 |
| 27 | 7.070239 | 0.370254 | 7.982354 | 0.202022 | 4.112577 | 0.212976 | 6.656074 | 0.169348 |
| 28 | 8.522865 | 0.378079 | 8.118445 | 0.1662 | 6.618646 | 0.126885 | 9.245152 | 0.162158 |
| 29 | 18.26104 | 0.017021 | 17.28405 | 0.158461 | 19.89989 | 0.243814 | 21.48232 | 0.604226 |
| 30 | 27.60778 | 0.555887 | 24.09741 | 0.525412 | 31.58188 | 0.845816 | 25.99251 | 0.441162 |
| 31 | 20.1486 | 0.700181 | 16.225 | 0.513608 | 22.55856 | 0.71903 | 14.50036 | 0.124691 |
| 32 | 7.287115 | 0.159424 | 5.867007 | 0.257745 | 8.251718 | 0.507595 | 5.238803 | 0.055502 |

*, percentage; **, standard error
