# Supplementary material for: HENMT1 and piRNA Stability Are Required for Adult Male Germ Cell Transposon Repression and to Define the Spermatogenic Program in the Mouse
Source: PLoS Genet. 2015 Oct 23;11(10):e1005620. doi: 10.1371/journal.pgen.1005620 (PMC4619860; doi:10.1371/journal.pgen.1005620)
Supplement: S3 Table — (DOCX) [file pgen.1005620.s003.docx]

**Table S3: Custom made Taqman probes and primers used for qPCR.**

|  | Forward primer | Reverse primer | Probe |
| --- | --- | --- | --- |
| *L1_A* | 5- AAGACCTCTGGTGAGTGGATCAC | 5- AAGACTCTGCTGGCAAGGTAGC | 5- CAAGTCCCTTCCGCTC |
| *L1_Tf14* | 5- GAGAGGGTGCGCCAGAGA | 5- TCAGCGCCTCTGTGCTTCT | 5- CCTGACAGCTTCTG |
| *Henmt1* transcript 3 | 5’-AAATGGAAATGGCAGAAAGCA | 5’-TTAGCAGCTTTAGGAGCTTGGC | 5’-CCGGTTGCCGACCT |
