# Supplementary material for: HENMT1 and piRNA Stability Are Required for Adult Male Germ Cell Transposon Repression and to Define the Spermatogenic Program in the Mouse
Source: PLoS Genet. 2015 Oct 23;11(10):e1005620. doi: 10.1371/journal.pgen.1005620 (PMC4619860; doi:10.1371/journal.pgen.1005620)
Supplement: S4 Table — (DOCX) [file pgen.1005620.s004.docx]

**Table S4: Primers for ChIP-qPCR.**

| **Primers** | **Sequences** |
| --- | --- |
| *Tnp1* Fw1 | TGCCATGAGTCTTTAGCTTGC |
| *Tnp1* Rev1 | GGTGTAGCAAAGGTTAGTGATGTC |
| *Tnp1* Fw2 | GACATCACTAACCTTTGCTACACC |
| *Tnp1* Rev2 | ACAGGAGTGGAGGAAGTTAGATG |
| *Tnp2* Fw1 | GGAGTCCCCACAGCCCCT |
| *Tnp2* Rev1 | CAGCACAATCTGACCTGACCC |
| *Tnp2* Fw2 | GGGTCAGGTCAGATTGTGCTG |
| *Tnp2* Rev2 | CTCTGTTACTTCTCCTGGCGTTGA |
| *Prm1* Fw1 | CAACTCCTGATGCCAAAGCC |
| *Prm1* Rev1 | GCACCCTCTTCCTCTTATAGATACT |
| *Prm1* Fw2 | GGCACTTAACACCTAAGCTGA |
| *Prm1* Rev2 | CAGTGAGGACCTGTACCATGT |
| *Prm2* Fw1 | ACTGGGCAGGGTGGGAACAA |
| *Prm2* Rev1 | GATTGGAGGAGGAGGGACGA |
| *Prm2* Fw2 | AAAGCAAGATGAGTAACTTGGC |
| *Prm2* Rev2 | CCAGGAGATCAGGAGGTTCTG |
| *Gapdhs* Fw1 | GCAATGTGACATCATCAGAAGC |
| *Gapdhs* Rev1 | ACAACAGTAACATTGGTAAGGAC |
| *Gapdhs* Fw2 | GCTTCTGTGTGAACCTAGTGGG |
| *Gapdhs* Rev2 | GCTTCTGATGATGTCACATTGC |
| *Ppia* Fw1 | TGTTTGCCTCAGTTACTTGCTC |
| *Ppia* Rev1 | GCTCCTACTAGATGGTTGCGG |
| *Ppia* Fw2 | ACTCTTCCCCCACCGACCG |
| *Ppia* Rev2 | TCACAACTGCTTTCCAAACG |
