# Supplementary figures and images for: HENMT1 and piRNA Stability Are Required for Adult Male Germ Cell Transposon Repression and to Define the Spermatogenic Program in the Mouse
Source: PLoS Genet. 2015 Oct 23;11(10):e1005620. doi: 10.1371/journal.pgen.1005620 (PMC4619860; doi:10.1371/journal.pgen.1005620)

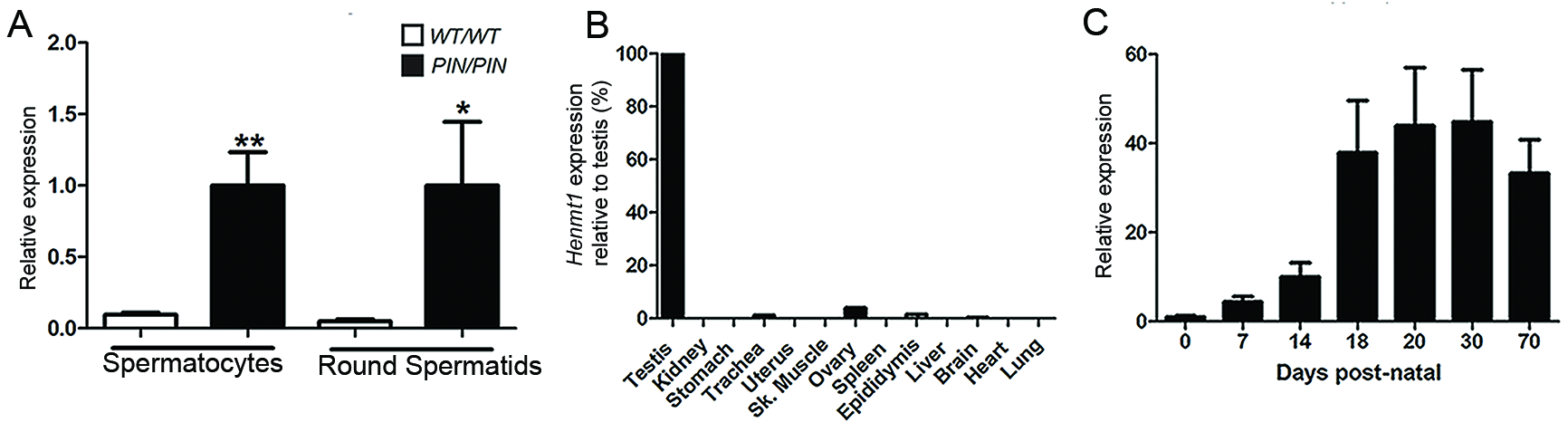

Supplement: S1 Fig — (A) Quantitative real-time PCR analysis for Henmt1 isoform 3 in 8 week old Henmt1 PIN/PIN and Henmt1 WT/WT testis. Values are relative to wild type testis (n = 3 / genotype +/- SD) (p*<0.05, ** p<0.01, mean ± SD). Two-tailed unpaired student T test was performed for statistical analyses. (B) qPCR analysis for Henmt1 mRNA in tissues (all isoforms). Values are relative to wild type testis (n = 3 / genotype +/- SD). (C) Henmt1 mRNA expression during the establishment of the first wave of spermatogenesis relative to day 0 expression (all isoforms, n = 3/ genotype). (TIF) [file pgen.1005620.s006.tif]

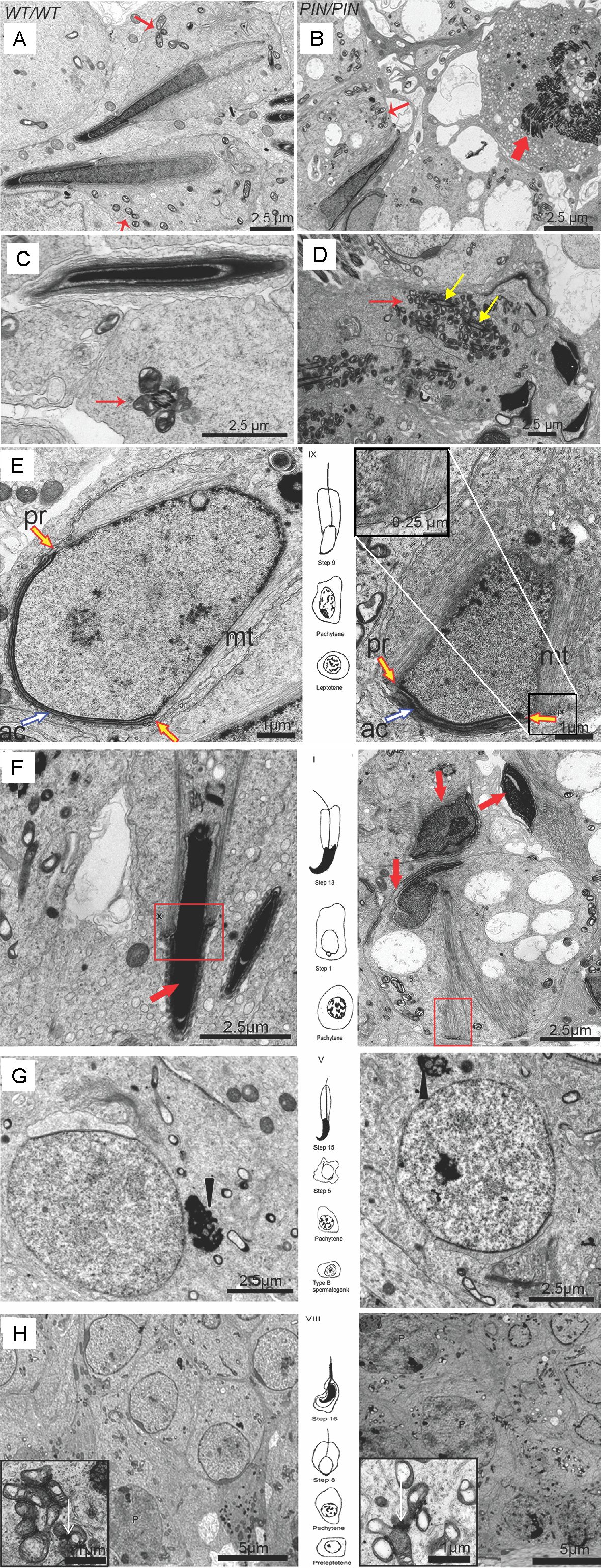

Supplement: S2 Fig — Electron microscopy demonstrating the presence of mitochondria in (A) Henmt1 WT/WT and (B) Henmt1 PIN/PIN elongating spermatids. Red thin arrows represent normal mitochondria distribution. Red thick arrows represents abnormal mitochondria clustering. (C) Step 16 Henmt1 WT/WT spermatids showing a well-organized mitochondrial sheath (red arrow) around the axoneme of the tail (yellow arrow). (D) Step 16 Henmt1 PIN/PIN spermatids with aggregated mitochondria and a poorly organized mitochondrial sheath around the axoneme (yellow arrow). (E) A stage IX tubule. In Henmt1 WT/WT (left hand side for the remainder of panels), step 9 spermatids containing normal machettes structures composed of a perinuclear ring (yellow arrow) adjacent to the caudal end of developing acrosome (ac) and an associated fringe of microtubules (mt). Step 9 Henmt1 PIN/PIN spermatids (right hand side in all panels) displayed abnormal manchette development. The perinuclear ring was orientated towards the plasma membrane. (F) A stage I Henmt1 WT/WT tubule containing step 13 spermatids showing a normal machette (red box) and condensed spermatids (arrow). Henmt1 PIN/PIN step 13 spermatids frequently contained ectopically placed machettes (red box) and heterogenous levels of chromatin condensation (arrows). (G) A Henmt1 WT/WT step 5 (stage V) spermatids containing normal chromatoid bodies composed of filaments that formed an irregular network of electron dense cords (arrowhead). Step 5 Henmt1 PIN/PIN spermatids contained similar, normal, intact chromatoid bodies (arrowhead). (H) Henmt1 WT/WT and Henmt1 PIN/PIN pachytene spermatocytes contained comparable inter-mitochondrial cement structures (stage VIII) (white arrow). Cartoons represent the germ cell types present within the seminiferous epithelium at the stage being analyzed. (TIF) [file pgen.1005620.s007.tif]

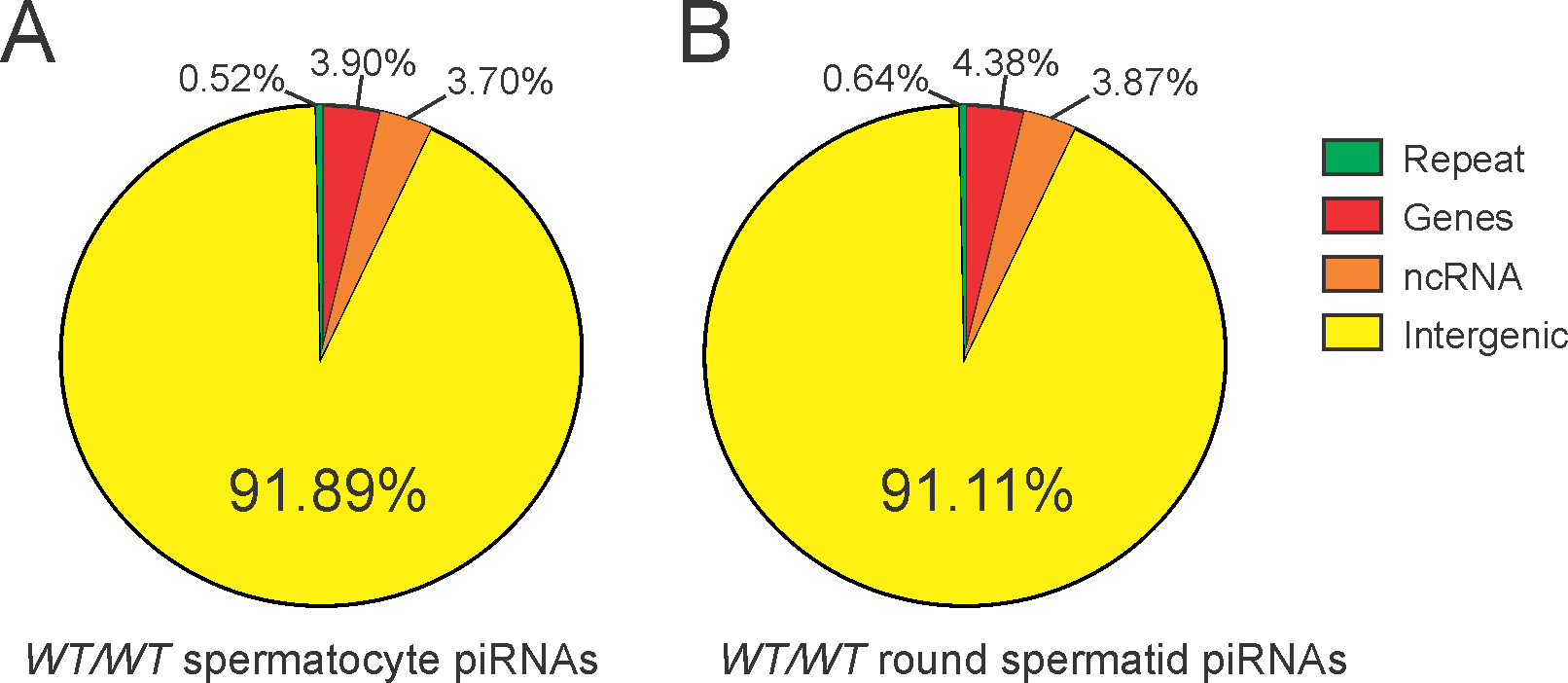

Supplement: S3 Fig — The percentage piRNAs classification in Henmt1 WT/WT spermatocytes (A) and round spermatids (B) from deep sequencing analyses. (TIF) [file pgen.1005620.s008.tif]

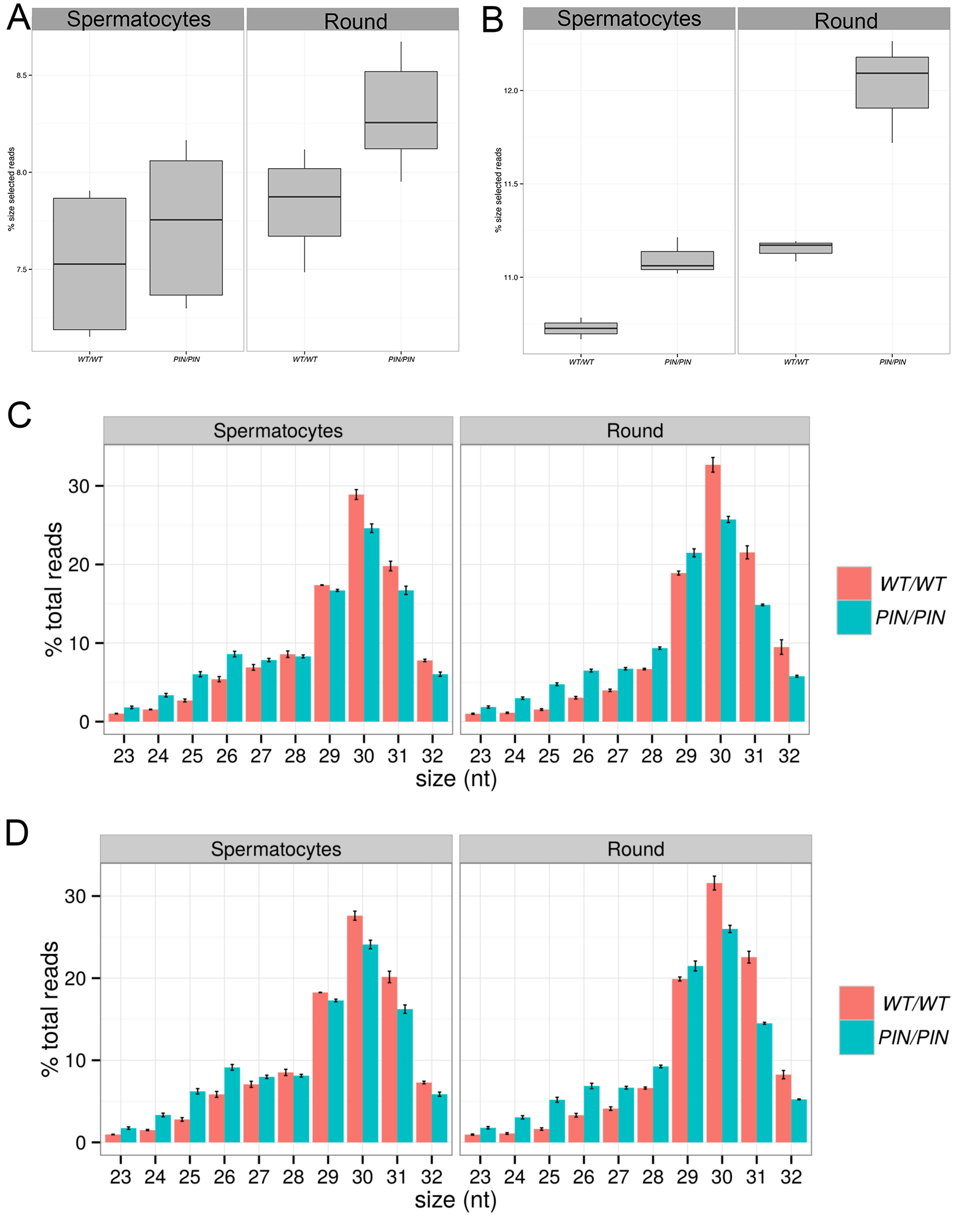

Supplement: S4 Fig — Boxplots showing the percentage of size selected reads defined as MILI (A) and MIWI-loaded (B) piRNAs in purified spermatocytes and round spermatids by overlapping with coordinates from HITS-CLIP data derived from whole mouse testis [33]. (C-D) The size distribution of MILI-loaded (C) and MIWI-loaded piRNAs (D), as defined by HITS-CLIP data [33], from Henmt1 WT/WT and Henmt1 PIN/PIN spermatocytes and round spermatids. These panels demonstrate the shifting of the length distribution of piRNAs to the left in Henmt1 PIN/PIN compared to Henmt1 WT/WT that results from end truncation. Please see S2 Table for the actual data used to plot the figure. (TIF) [file pgen.1005620.s009.tif]

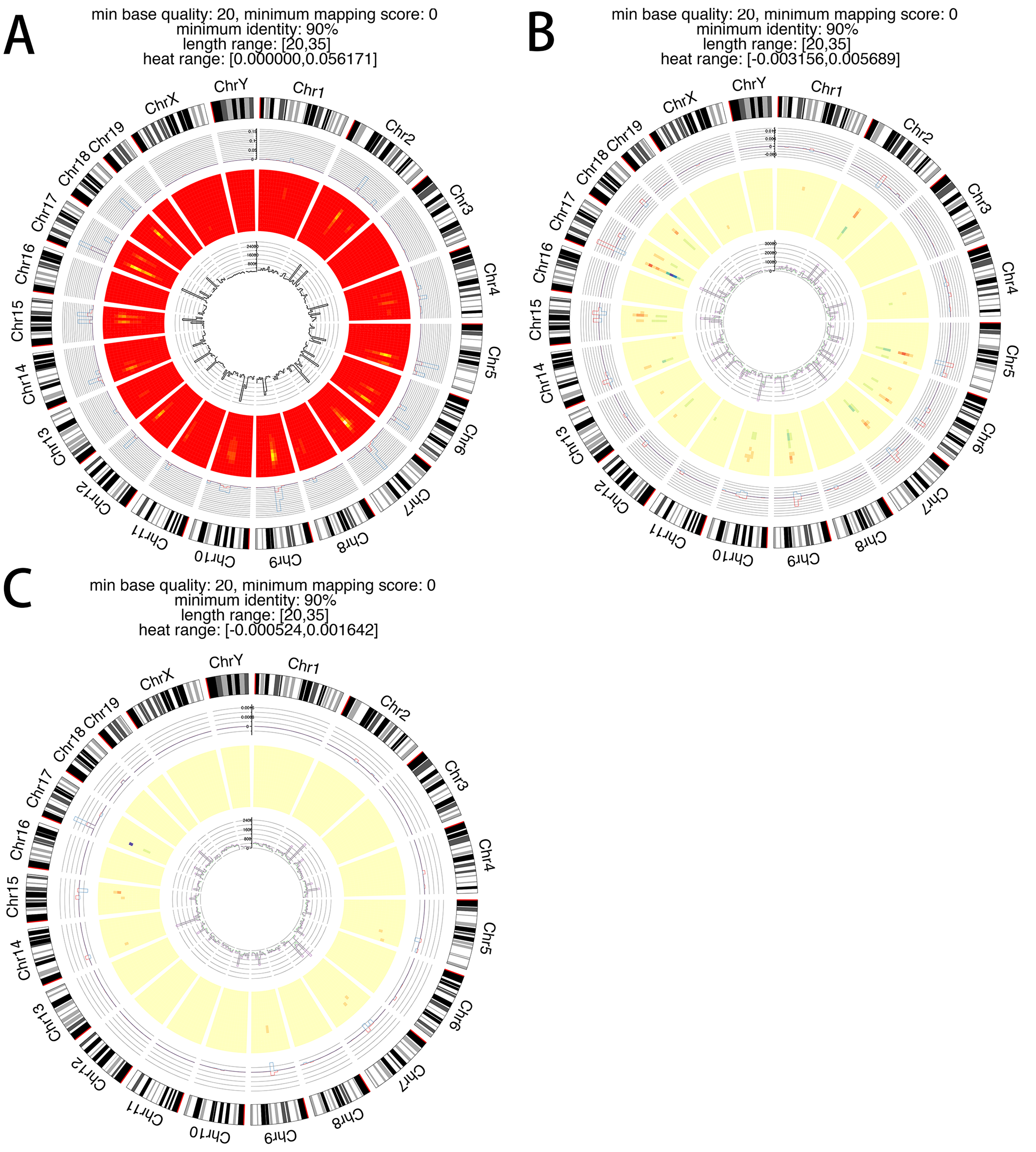

Supplement: S5 Fig — (A) Global expression of primary piRNA species in spermatocytes was significantly localized to a small number of well-defined loci. A representation of mapping frequency of cutadapt-trimmed primary piRNA sequences to 10Mbp bins of the mouse genome. The heat map shows piRNA mapping counts per base pair with each bin stratified by piRNA length from 20 bp (inner edge) to 35 bp (outer edge) inclusive. The outer trace track shows the summed piRNA mapping counts per base for long (28-32bp inclusive; blue) and short (23-27bp inclusive; red) piRNA species. The inner trace track shows the number of distinct 5' ends mapped to each bin location. (B) piRNAs in Henmt1 PIN/PIN germ cells were shortened. Differential expression analysis of primary piRNA species in spermatocytes showed an overall reduction in Henmt1 PIN/PIN cells of long piRNA species and an increase of short piRNA species. A representation of differential mapping frequency of all cutadapt-trimmed primary piRNA sequences mapped to 10Mbp bins of the mouse genome. The heat map shows differential piRNA mapping counts (mutant vs wild type) per base pair in each bin stratified by piRNA length from 20 bp (inner edge) to 35 bp (outer edge) inclusive. There is a clear deficit of longer piRNAs and clear excess of shorter piRNAs in mutant spermatocytes as evidenced by this result. The outer trace track shows the summed change in piRNA mapping counts per base for long (28-32bp inclusive; blue) and short (23-27bp inclusive; red) piRNA species. The inner trace track shows the number of distinct 5' ends mapped to each bin for wild type (green) and mutant (magenta). A description of the analysis strategy is provided in the Materials and Methods. (C) The expression of TE-directed piRNAs in Henmt1 mutant spermatocytes. Differential expression analysis of L1-targeted primary piRNA species (piRNAs with sequence similarity to L1 elements) in spermatocytes showed an increase in Henmt1 PIN/PIN cells of long piRNA species at the chromoso [file pgen.1005620.s010.tif]

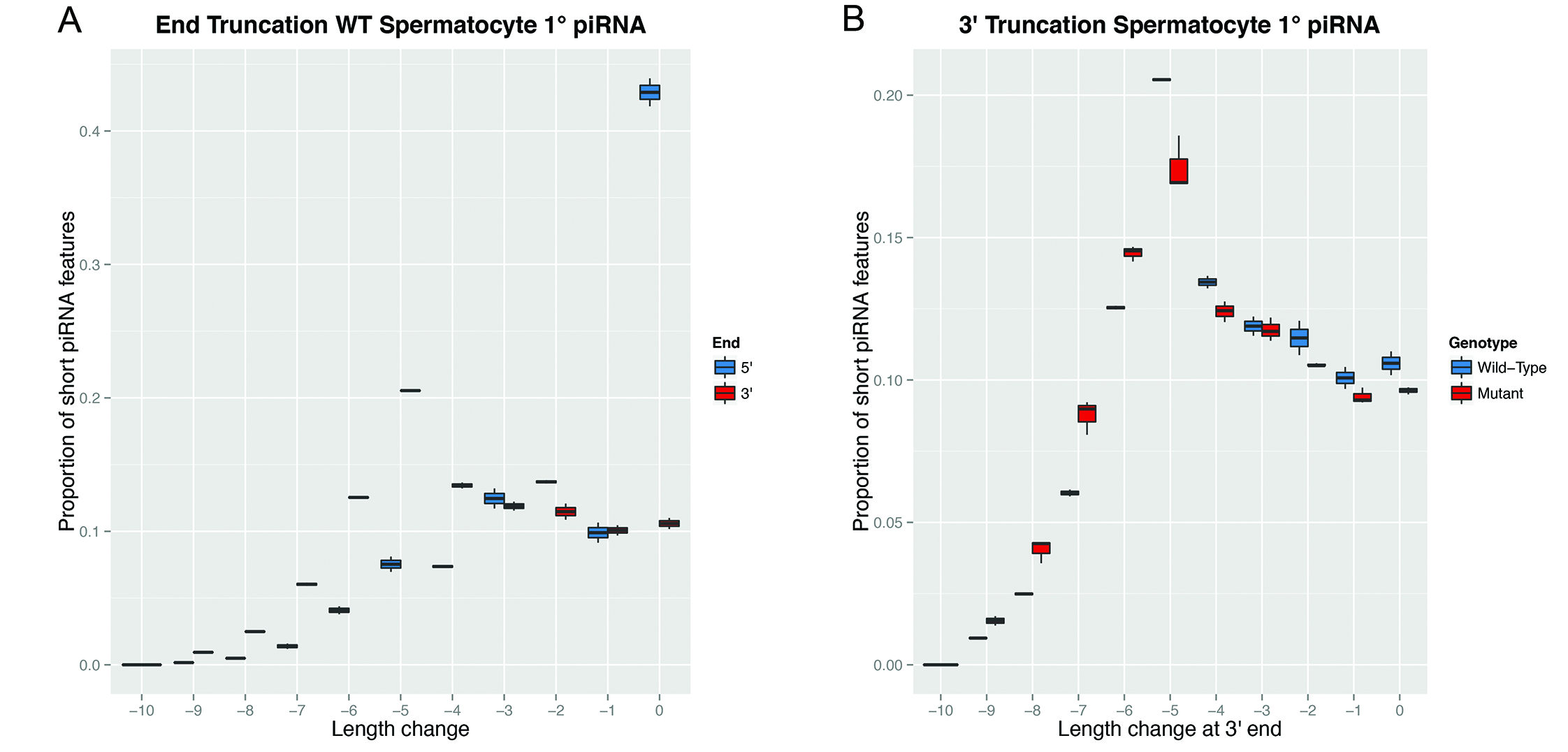

Supplement: S6 Fig — Quantification of truncation of primary piRNA ends from spermatocytes represented by end-length change where negative length change signifies truncation. (A) Proportions of primary piRNA populations from wild type spermatocyte tissue showing 5' (blue) and 3' (red) end truncation. There was no significant increase in the truncation of piRNA at the 5' end compared to the 3' end. Longer truncations (four bases or more) are significantly more frequent at the 3' end than at the 5' end. Results for secondary piRNA and samples taken from round spermatids showed qualitatively similar patterns. A description of the analysis strategy is provided in the Materials and Methods. The 95% confidence interval for the extent of 3' end truncation compared to 5' end truncation: wild type spermatocyte 1°, 1.866477–1.872112; wild type spermatid 1°, 1.904809–1.913252; wild type spermatocyte 2°, 2.128158 2.139266; wild type spermatid 2°, 2.203954 2.217913; mutant spermatocyte 1°, 2.024495–2.028101; mutant spermatids 1°, 2.014840–2.019387; mutant spermatocyte 2°, 2.413812–2.421914; mutant spermatids 2°, 2.371488–2.379345). (B) Proportions of primary piRNA populations from wild type (blue) and mutant (red) spermatocytes showing 3' end truncation. Small (five or fewer bases) length changes were elevated in wild type compared to mutant while larger length changes were elevated in mutant. The 95% confidence intervals for change in 3' end truncation in Henmt PIN/PIN: spermatocyte 1°, 0.2749190–0.2798546; spermatid 1°, 0.2761497–0.2832534; spermatocyte 2°, 0.2835850–0.2939058; spermatid 2°, 0.2756250–0.2877403). (TIF) [file pgen.1005620.s011.tif]

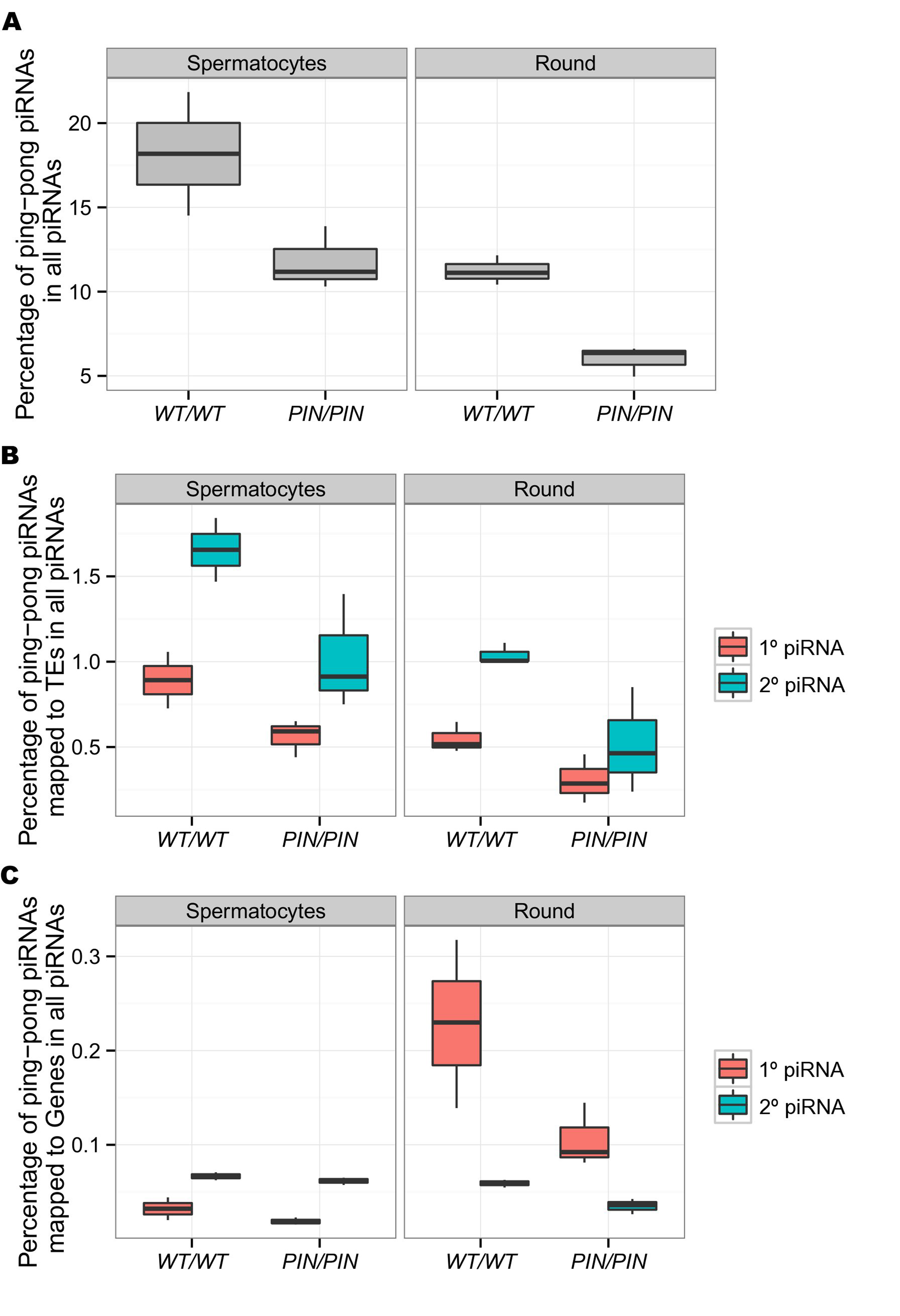

Supplement: S7 Fig — Stringent ping-pong cycle derived piRNAs were defined by the following criteria: 1) size from 23-32nt; 2) 1st base of primary (1°-piRNA) piRNA is “U” or 10th base of secondary (2°-piRNA) piRNA is “A”; 3) primary (1°-piRNA) piRNA should have 10 nt overlap at the 5’ end with its corresponding secondary (2°-piRNA) piRNAs at the 5’ end; 4) primary (1°-piRNA) piRNA should be mapped to repeats or protein-coding RNAs in sense and secondary (2°-piRNA) piRNA should be mapped to repeats or protein-coding RNAs in antisense. (A) Proportion of ping-pong derived piRNAs in all piRNAs. (B) Proportion of primary (1°-piRNA) or secondary (2°-piRNA) ping-pong piRNAs mapped to all repeats in all piRNAs. (C) The proportion of primary (1°-piRNA) or secondary (2°-piRNA) ping-pong piRNAs mapped to all protein-coding genes in all piRNAs. (TIF) [file pgen.1005620.s012.tif]

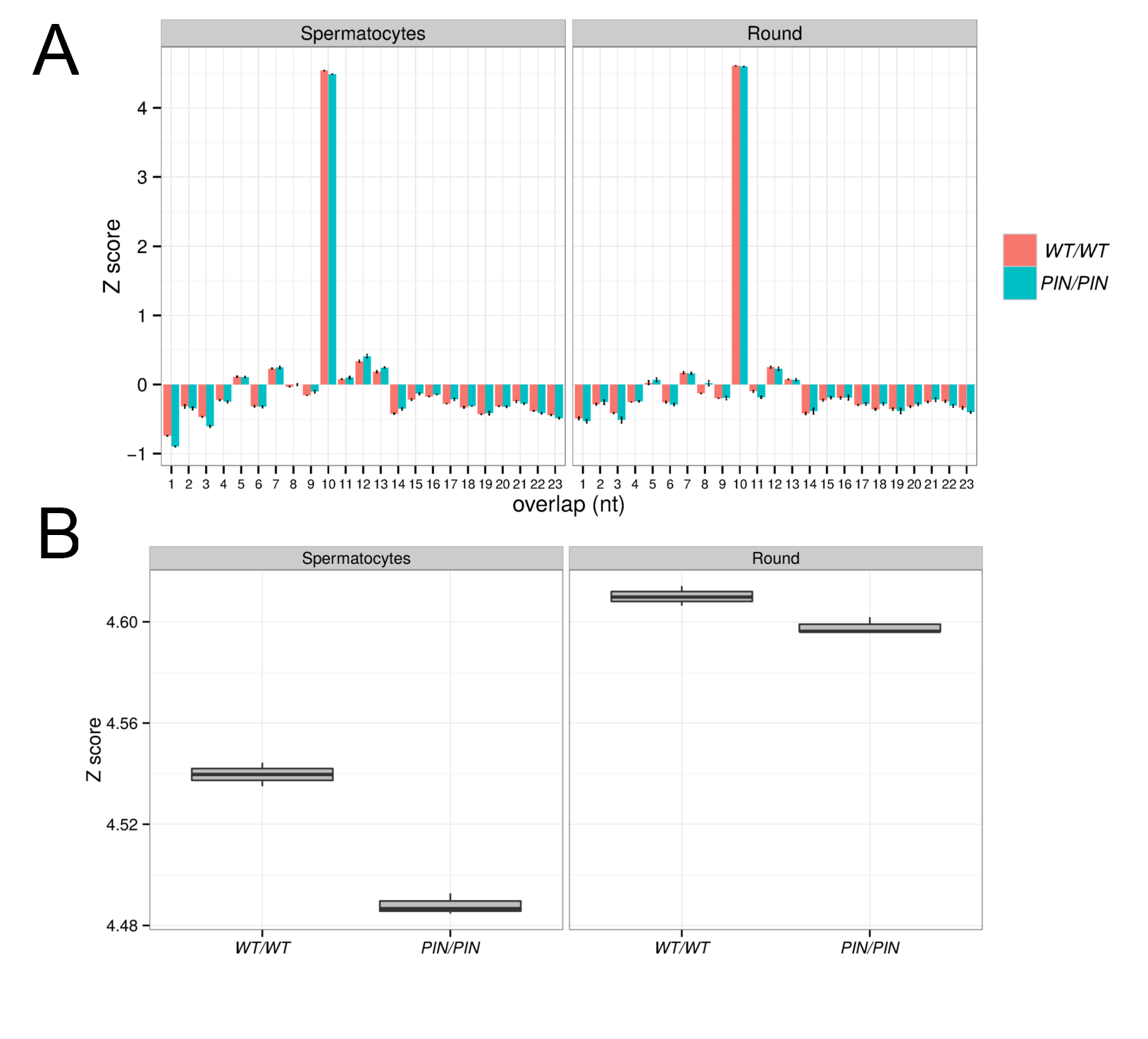

Supplement: S8 Fig — (A) Z scores of the different overlapping lengths of piRNA pairs from 5’ end showed biased 10 nt overlap, which is a signature of ping-pong piRNA amplification. (B) Decreased ping-pong z scores were observed in both Henmt1 PIN/PIN spermatocytes and round spermatids compared to Henmt1 WT/WT. (TIF) [file pgen.1005620.s013.tif]

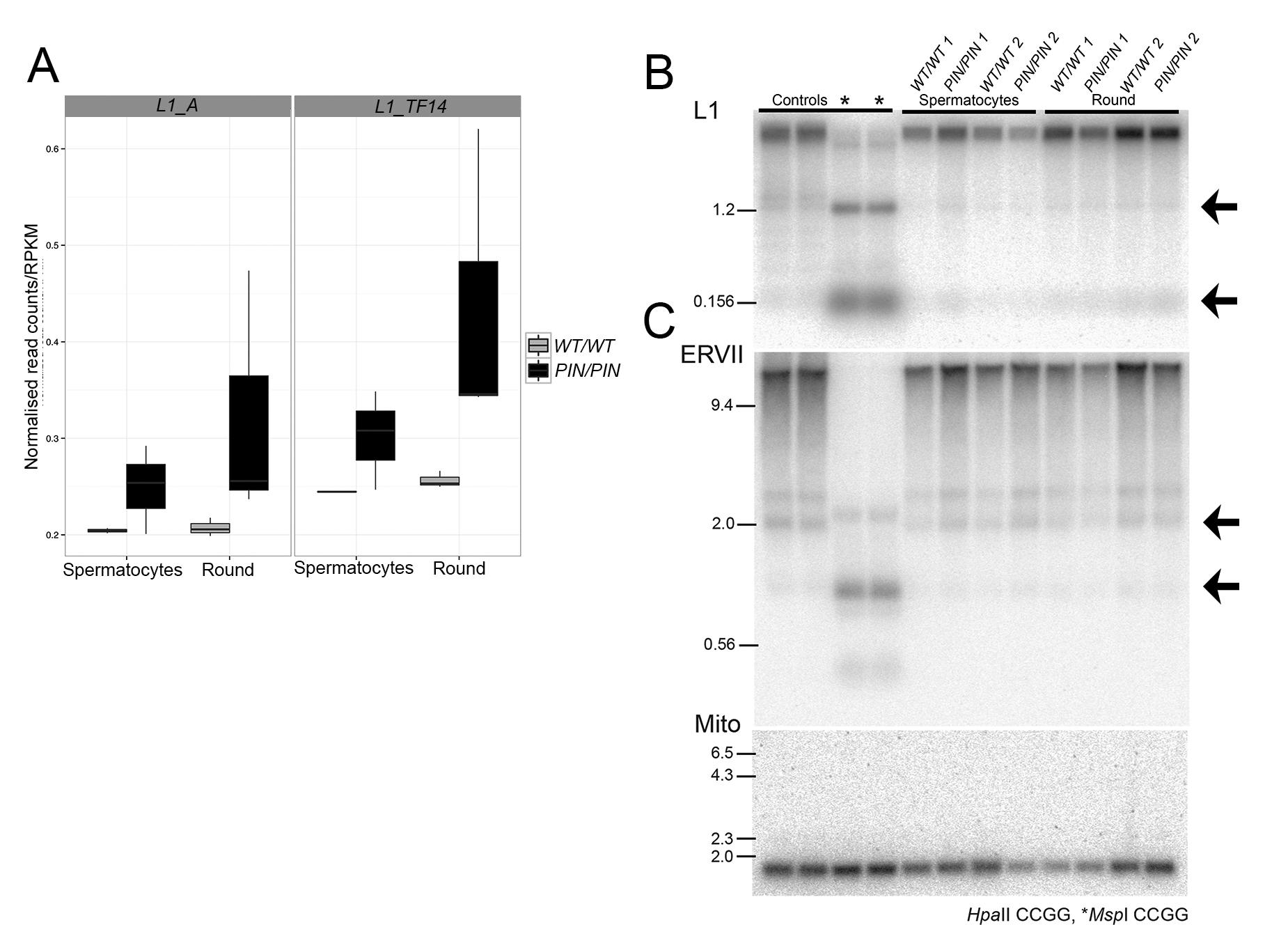

Supplement: S9 Fig — (A) Transcriptome analysis of Henmt1 WT/WT and Henmt1 PIN/PIN spermatocytes and round spermatids demonstrated increased L1_A and L1_TF14 expression in the Henmt1 PIN/PIN spermatocytes (PS) and round spermatids (RS). (B-C) gDNA was digested with methylation sensitive enzymes (HpaII) and methylation insensitive enzyme (MspI) * gDNA digested with MspI, (B) Southern blot was hybridized with L1 probe (C) with an ERVII probe. Mito probe is used for loading control. The arrow indicates the presence of methylation sensitive restriction products in the control testis. (TIF) [file pgen.1005620.s014.tif]

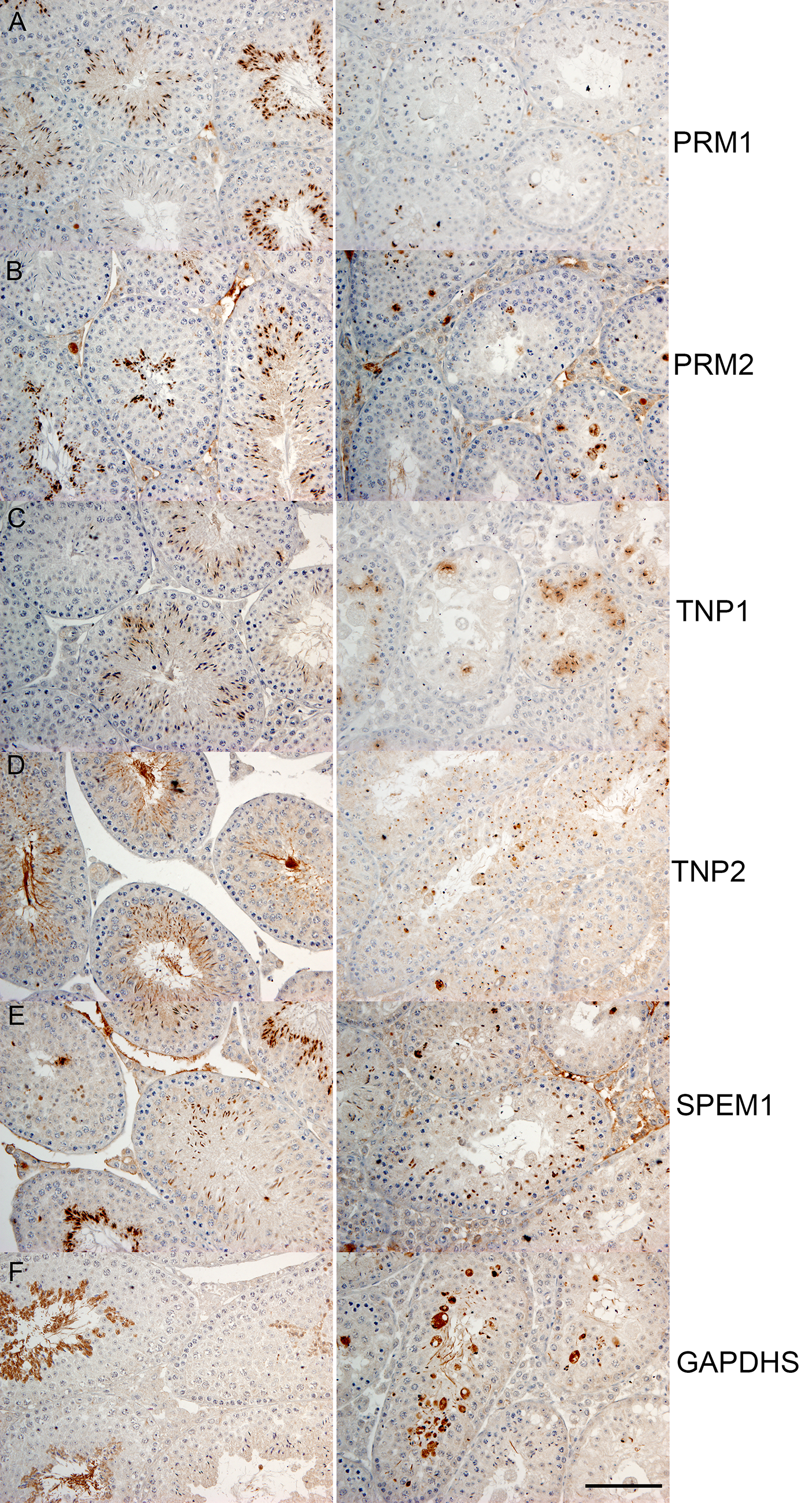

Supplement: S10 Fig — Immunostaining for of key spermatid proteins in Henmt1 WT/WT (left panel) and Henmt1 PIN/PIN (right panel) in full spermatogenesis. (A) PRM1, (B) PRM2, (C) TNP1, (D) TNP2, (E) SPEM1 and (F) GAPDHS. Scale bar = 100μm. (TIF) [file pgen.1005620.s015.tif]
